# Hydrogen sulfide as a new therapeutic target of pulmonary hypertension: an overview with update on immunomodulation

**DOI:** 10.3389/fphar.2025.1510275

**Published:** 2025-05-12

**Authors:** Xue-Xue Zhu, Chen-Yang Zhao, Qing-Bo Lu, Ao-Yuan Zhang, Xin-Yu Meng, Jia-Bao Su, Guo Chen, An-Jing Xu, Hai-Jian Sun, Xiao-Wei Nie

**Affiliations:** ^1^ MOE Medical Basic Research Innovation Center for Gut Microbiota and Chronic Diseases, School of Medicine, Jiangnan University, Wuxi, China; ^2^ Department of Endocrinology, Affiliated Hospital of Jiangnan University, Jiangnan University, Wuxi, Jiangsu, China; ^3^ Department of Pharmacology, School of Medicine, Southern University of Science and Technology, Shenzhen, Guangdong, China

**Keywords:** hydrogen sulfide, pulmonary hypertension, oxidative stress, immunomodulation, organosulfur compounds

## Abstract

Pulmonary hypertension (PH) is a complex and progressive vascular disease characterized by elevated pulmonary arterial pressure (PAP) and vascular resistance, leading to right ventricular failure and, ultimately, death. Current therapies primarily focus on vasodilation and symptom management, but there remains a critical need for treatments that address the underlying pathophysiological mechanisms of PH. Numerous studies have identified hydrogen sulfide (H_2_S) as a potential therapeutic target in PH. Traditionally recognized for its toxic effects at high concentrations, H_2_S is now known to play crucial roles in various physiological processes, including vasodilation, anti-inflammation, and antioxidation, which are relevant to PH pathogenesis. Given its multifaceted roles in the pathophysiology of PH, H_2_S represents a promising therapeutic target. Strategies to enhance endogenous H_2_S production or administer exogenous H_2_S donors are being explored as potential treatments for PH. These approaches aim to harness the vasodilatory, anti-inflammatory, antioxidant, and anti-remodeling properties of H_2_S to mitigate disease progression and improve patient outcomes. Future research should focus on optimizing H_2_S-based therapies and exploring their clinical efficacy and safety in PH patients.

## Introduction

Pulmonary hypertension (PH) has a prevalence of 11–26 cases per million adults, with a mortality rate of approximately 50% at 5 years despite targeted therapies (Thenappan et al., 2018). PH is a rare disorder marked by remodeling of small pulmonary arteries and increased pulmonary arterial pressure (PAP), leading to progressive right heart failure and death (Austin and Loyd, 2014; Zhang and Sun, 2020a; Zhang et al., 2023; Zhang et al., 2024). This remodeling involves interactions among various cell types, including endothelial cells, smooth muscle cells, and fibroblasts, across the distinct layers of the pulmonary arteries, resulting in histological alterations to the vessel wall (Zhang and Sun, 2021; 2022; Bian and Chen, 2024). Endothelial dysregulation and proliferation in the intima, smooth muscle cell proliferation and resistance to apoptosis in the medial layer, and adventitial fibroblast activation collectively contribute to the narrowing of the vascular lumen, thus causing PH (Nie et al., 2020; Wołowiec et al., 2023). Consequently, strategies that inhibit cell proliferation or induce apoptosis to reverse pulmonary remodeling may offer therapeutic potential for PH. Emerging evidence suggests that PH shares numerous pathogenic mechanisms with cancers, positioning it as a proliferative disorder with cancer-like characteristics (Sun H. J. et al., 2022; He et al., 2023). This similarity presents opportunities to explore cancer-specific therapeutic approaches, including the use of anti-cancer agents, for the treatment of PH (Nicoleau et al., 2021; Li et al., 2022).

PH is classified into four groups, including pulmonary arterial hypertension (PAH), PH due to left heart disease, PH due to lung diseases or hypoxia, and PH due to pulmonary artery obstruction (Castillo-Galán et al., 2025; Hu et al., 2025). PAH is found to result from endothelial dysfunction, vasoconstriction, and vascular remodeling of the small pulmonary arteries. Elevated left atrial pressure due to systolic or diastolic dysfunction, valvular disease, or congenital defects leads to pulmonary venous hypertension and passive pulmonary artery pressure elevation. Chronic hypoxia causes pulmonary vasoconstriction, leading to vascular remodeling. Conditions include chronic obstructive pulmonary disease (COPD), interstitial lung disease, and sleep apnea. Chronic thromboembolic pulmonary hypertension (CTEPH) results from unresolved pulmonary emboli and thrombus organization, leading to mechanical obstruction and secondary remodeling. The etiopathogenesis of PH involves a complex interplay of genetic, molecular, and environmental factors, leading to vascular remodeling, increased pulmonary vascular resistance, and right ventricular dysfunction. Specifically, the pathogenesis of PH is driven by factors such as mutations in the type II bone morphogenetic protein receptor (BMPR2), chronic inflammation, fibrosis, immune activation, and mitochondrial metabolic dysfunction (Thenappan et al., 2018).

The current management of PH aims to improve symptoms, delay disease progression, and reduce mortality (Ge et al., 2024). Treatment strategies vary depending on the PH group and disease severity. Generally, oxygen therapy for hypoxia, diuretics for volume overload, physical activity, and pulmonary rehabilitation are routine methods for treating PH. In addition, endothelin receptor antagonists (Bosentan, Ambrisentan, and Macitentan), phosphodiesterase-5 inhibitors including Sildenafil, and Tadalafil, soluble guanylate cyclase (sGC) stimulator Riociguat, prostacyclin analogues and prostacyclin receptor agonists including Epoprostenol, Treprostinil, ans Selexipag, calcium channel blocker including Nifedipine, and Amlodipine are commonly used drugs for treating PH (Azaredo Raposo et al., 2024; DeVaughn et al., 2024; Madonna et al., 2024; Tangcharoen et al., 2024; Doktor et al., 2025; Yi et al., 2025). Overall, PH treatment requires a multidisciplinary approach and close monitoring to optimize outcomes.

The immune system plays a significant role in the pathogenesis of PH, with various immune cells, cytokines, and signaling pathways contributing to vascular remodeling, inflammation, and fibrosis in the pulmonary vasculature (Zhao et al., 2024). Overproduction of inflammatory cytokines and chemokines, immune cell infiltration, and NLRP3 inflammasome activation are critically implicated in the pathogenesis of PH (Rudyk and Aaronson, 2021). Understanding the immune mechanisms in PH has led to the exploration of immunomodulatory therapies. Drugs targeting specific cytokines, chemokines, or immune cells, as well as those modulating the NLRP3 inflammasome, are being investigated for their potential to treat PH. PH exhibits heterogeneous and multifactorial pathophysiology, underscoring the importance of a comprehensive understanding of their underlying mechanisms. Despite existing treatments, including renin-angiotensin system blockers, calcium antagonists, steroidal mineralocorticoid receptor antagonists, and thiazide-type diuretics, nearly half of PH patients fail to achieve adequate control (Halimi et al., 2024). Thus, optimizing the prevention, treatment, and diagnosis of PH necessitates a deeper exploration of its pathogenic network.

**FIGURE 1:**
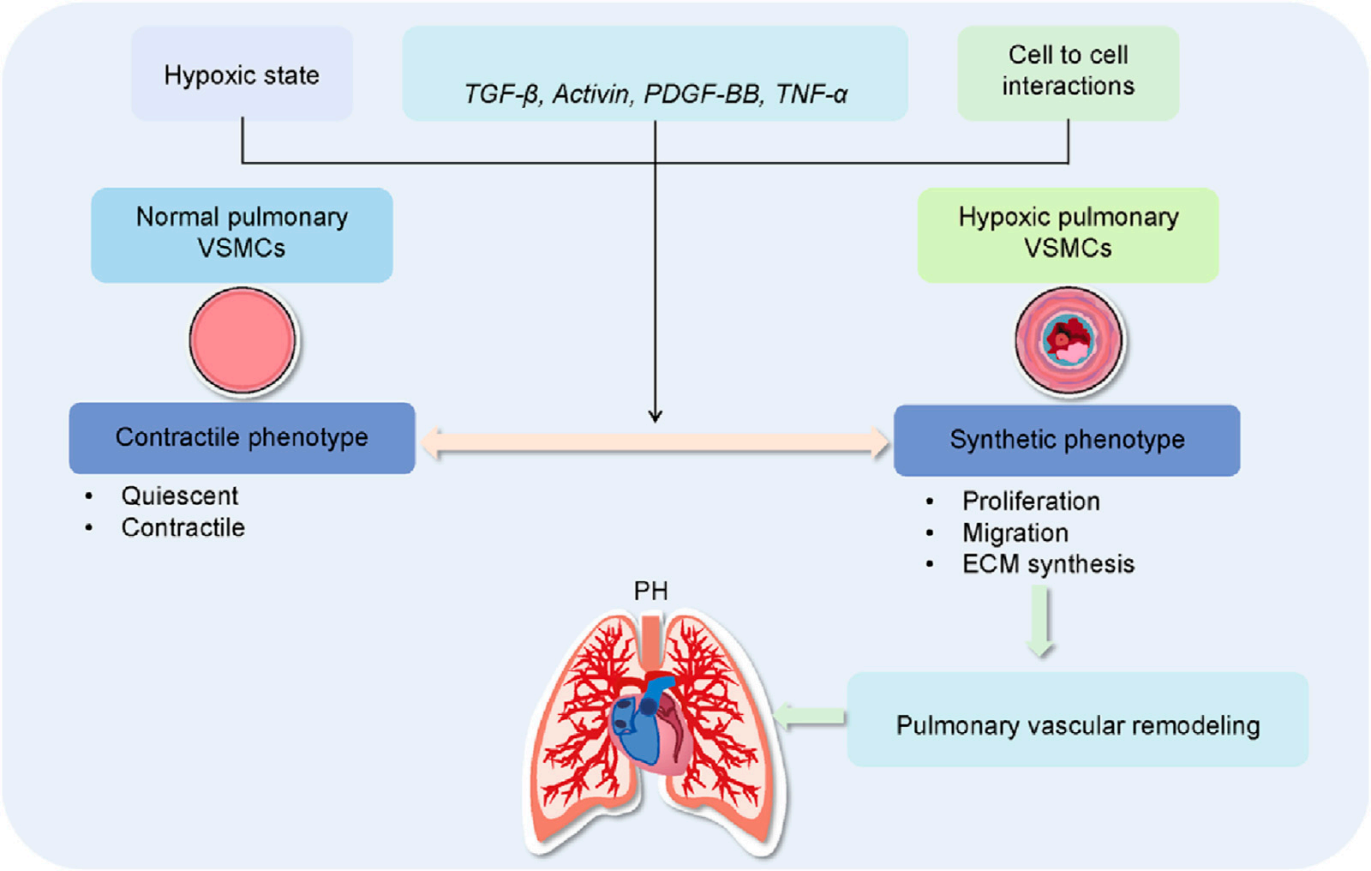
Role of VSMC dysfunction in pulmonary vascular remodeling and PH. In the pulmonary circulation, the heterogeneity and phenotypic plasticity of vascular smooth muscle cells (VSMCs) are crucial for maintaining vascular function in the lungs. Quiescent pulmonary VSMCs exhibit a contractile phenotype, characterized by low extracellular matrix (ECM) synthesis and a fusiform shape, representing their differentiated state. In response to hypoxia, growth factors, inflammatory mediators, pulmonary VSMCs transition from a contractile to a synthetic phenotype. This change is marked by increased proliferation, migration, and ECM synthesis, along with the development of PH.

The endogenous gasotransmitters, such as nitric oxide (NO), carbon monoxide (CO), hydrogen sulfide (H_2_S), and sulfur dioxide (SO_2_), are characterized by their rapid generation, swift transmission, diverse functions, and short half-lives, playing pivotal roles in the pathogenesis of PH (Huang et al., 2022; Sun Y. et al., 2022; Liu et al., 2023; Gerges et al., 2024; Madonna et al., 2024; Kanemaru and Ichinose, 2025; Zhang et al., 2025). Significant progress has been made in elucidating the involvement of these gasotransmitters in PH development (Sun Y. et al., 2022; Song et al., 2024). Among them, H_2_S, the third identified gasotransmitter after NO and CO, is particularly implicated in various cardiovascular diseases, including PH (Turhan et al., 2022; Wang and Tang, 2022). A downregulated endogenous H_2_S pathway has been observed in pulmonary vascular diseases such as PH (Zhang et al., 2003; Chen et al., 2020; Roubenne and Marthan, 2021). However, exogenous H_2_S donors can mitigate the progression of PH (Chunyu et al., 2003; Schiliro et al., 2021). H_2_S is involved in the regulation of vascular tone, reduction of blood pressure and PAP, inhibition of vascular smooth muscle cell (VSMC) proliferation, modulation of endothelial inflammatory responses, induction of VSMC apoptosis, and prevention of vascular collagen remodeling (Figure 1) (Qingyou et al., 2004). Notably, H_2_S exerts several immune regulatory mechanisms that influence cardiovascular health, including anti-inflammatory, antioxidant, and cytoprotective effects (Pan et al., 2017). H_2_S regulates the activity of various immune cells, including macrophages, T Cells, and neutrophils. It can shift macrophages from a pro-inflammatory M1 phenotype to an anti-inflammatory M2 phenotype, thereby promoting tissue repair and reducing inflammation in the cardiovascular system. H_2_S is reported to decrease the adhesion of leukocytes to the endothelium, a critical step in the initiation of inflammation and atherosclerosis. By reducing leukocyte adhesion, H_2_S prevents the infiltration of immune cells into the vascular wall and subsequent inflammatory responses. H_2_S has been revealed to modulate the expression of chemokines and adhesion molecules (e.g., ICAM-1, VCAM-1) on endothelial cells, further controlling the recruitment and migration of immune cells into cardiovascular tissues.

This review will summarize recent advancements in understanding the functional and mechanistic roles of H_2_S in the inflammatory and immunoregulatory processes underlying PH. We will also discuss the crucial role of H_2_S in various PH subtypes, including hypoxic PH (HPH), monocrotaline (MCT)-induced PH, high blood flow-induced PH, congenital heart disease (CHD)-associated PH, and COPD-associated PH. Additionally, we address the potential challenges in developing H_2_S-based therapies to modulate pathological processes in PH. A deeper understanding of the immunomodulatory and biochemical functions of H_2_S may pave the way for novel therapeutic strategies targeting PH.

## H_2_S biosynthesis and catabolism

H_2_S is primarily produced endogenously through the enzymatic pathways, whereas the non-enzymatic pathways also contribute to its mobilization under physiological conditions (Olson, 2018; Yang et al., 2019). In physiological settings, approximately 28% of H_2_S exists in its undissociated form, 72% as hydrosulfide anions (HS^−^), and a negligible amount as sulfide anions (S_2-_) (Li and Lancaster, 2013). The effects of the undissociated and dissociated forms are collectively referred to as H_2_S. Initial studies estimated H_2_S concentrations at around 30 µM in lung tissue, increasing to over 200 µM in the heart, brain, and plasma (Furne et al., 2008). However, those methodological issues, such as variability in pH, temperature, and substrate concentration, indicated these levels might not reflect physiological reality (Vitvitsky et al., 2012). More accurate assessments suggest that steady-state H_2_S concentrations are in the nanomolar range in most tissues and plasma (Vitvitsky et al., 2012). This lower concentration aligns with the high turnover rate of H_2_S, characterized by significant production and rapid oxidation under physiological conditions (Vitvitsky et al., 2012). At concentrations exceeding 50 μM, H_2_S can have detrimental effects, including mitochondrial poisoning due to the inhibition of cytochrome c oxidase (complex IV) (Cooper and Brown, 2008; Libiad et al., 2019). Therefore, the dual effects of H_2_S necessitate careful regulation of its production and clearance.

**FIGURE 2:**
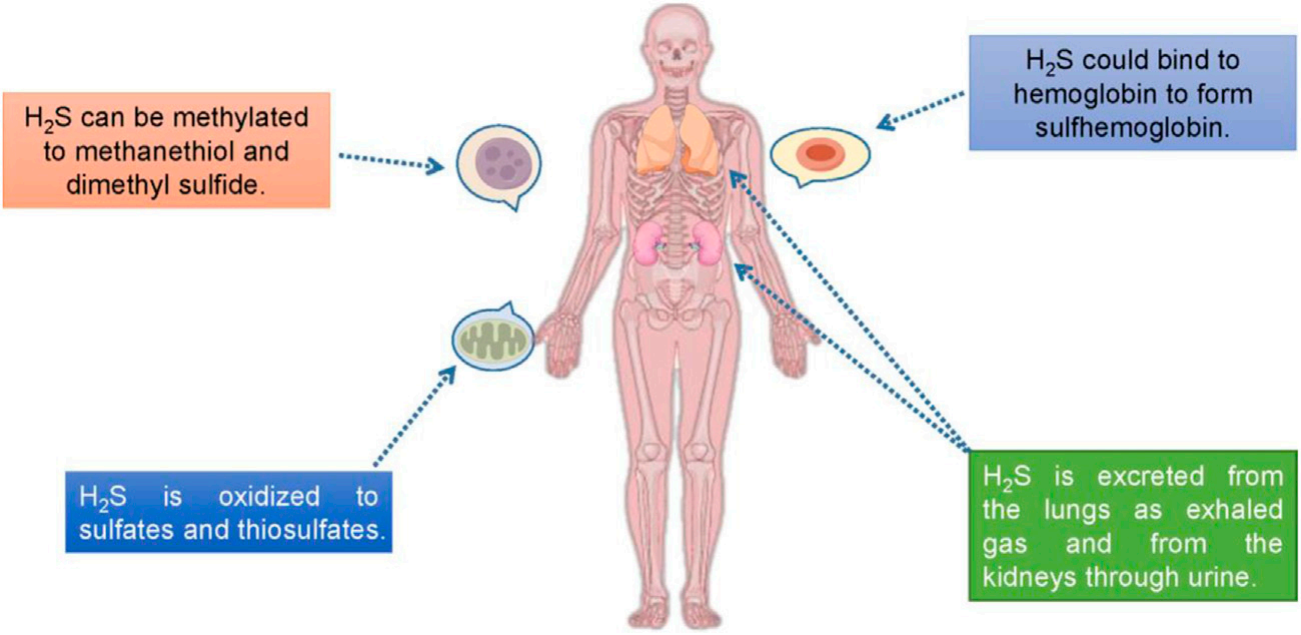
There are four known pathways for H_2_S decomposition in mammal system.

Traditionally, H_2_S is generated endogenously through both enzymatic and non-enzymatic pathways (Chiku et al., 2009). The primary enzymatic production occurs in the cytoplasm, where L-cysteine (L-Cys) serves as the main substrate. Three key enzymes involved in this process include cystathionine-γ-lyase (CSE), cystathionine-β-synthase (CBS), 3-mercaptopyruvate sulfur transferase (3-MST), and cysteine aminotransferase (CAT) (Chiku et al., 2009). CSE and CBS catalyze the production of H_2_S in the cytoplasm using L-cysteine and L-homocysteine as substrates (Majtan et al., 2018). Additionally, 3-MST, in conjunction with CAT, generates H_2_S from L-cysteine in both the cytoplasm and mitochondria. 3-MST can also produce H_2_S from D-cysteine in coordination with D-amino acid oxidase (Majtan and Kožich, 2023). The expression of these enzymes is tissue-specific: CSE is abundant in the thoracic aorta, liver, portal vein, ileum, and non-vascular tissues; CBS is predominantly found in the brain, kidney, and liver; and 3-MST is active in the aorta, kidney, brain, and liver (Li et al., 2011). CSE is particularly crucial for H_2_S production in the cardiovascular system. Recent advances in the study of endogenous H_2_S metabolism suggest that H_2_S can be generated independently of CSE, CBS, and 3-MST/CAT coupling pathways. In contrast to the enzymatic pathways, non-enzymatic H_2_S production is partially catalyzed by the synergistic action of vitamin B6 and iron, using cysteine as a substrate (Paul and Snyder, 2012). This non-enzymatic pathway is active in the heart, lungs, spleen, muscles, plasma, bone marrow, and especially in erythrocytes. Free H_2_S can be oxidized in mitochondria by sulfhydryl reductase (SQR) or methylated in the cytoplasm by sulfhydryl-S-methyltransferase (Hu and Lukesh, 2023). Additionally, H_2_S is excreted through biological fluids after binding with methemoglobin or other metal and disulfide-containing molecules (Figure 2). H_2_S levels can be increased using inorganic sulfide salts, organic H_2_S donors, or phosphodiesterase inhibitors. Common H_2_S donors include sodium hydrosulfide, GYY4137, 4-carboxyphenyl-isothiocyanate acid esters (4CPI), SG-1002, cysteine analogs, S-propylcysteine, S-allylcysteine, N-acetylcysteine, and drug chimeras such as l-DOPA, NOSH-sulindac (AVT-18A), NOSH-aspirin, and ACS67 (a combination of latanoprost and an H_2_S-releasing moiety) (Zhang L. et al., 2018). S-propargyl-cysteine (SPRC), also known as ZYZ-802, is a slow-releasing H_2_S donor and an analog of S-allylcysteine (SAC), which is abundant in aged garlic (*Allium sativum*) extract (Wen and Zhu, 2015). N-acetylcysteine (NAC) is widely used as an antioxidant and cell protectant (Cerda and Pluth, 2018), and L-cysteine is a substrate for endogenous H_2_S production (Salvi et al., 2016). Mitochondria-targeted compounds like AP39 and AP123 also play roles in H_2_S regulation (Gerő et al., 2016). Correspondingly, it is crucial to understand the role of H_2_S in the physiology and pathophysiology of pulmonary blood vessels by using exogenous H_2_S donors or regulating endogenous H_2_S. Although minor, non-enzymatic H_2_S release occurs through the chemical reduction of reactive sulfur groups in thiosulfates or polysulfides. Benavides et al. demonstrated that dietary *A. sativum*-derived organic polysulfides, such as diallyl disulfide and diallyl trisulfide, can release H_2_S in the presence of glutathione (GSH) and glucose metabolism (Benavides et al., 2007). Consequently, it has been suggested that basal circulating H_2_S levels may result from this non-enzymatic process.

H_2_S functions as a signaling molecule only at physiological, relatively low concentrations. At higher concentrations, H_2_S becomes toxic by inhibiting cytochrome c oxidase (complex IV) of the mitochondrial respiratory chain, impairing cellular respiration (Jiang et al., 2024; Nishimura et al., 2024). To prevent this toxicity, H_2_S bioavailability is tightly regulated through its safe storage as bound sulfane sulfur and/or efficient catabolism (Ichinose and Hindle, 2024). In mammals, H_2_S is primarily oxidized in mitochondria via the sulfide oxidation pathway, with thiosulfate and sulfate as the final products (Iciek and Bilska-Wilkosz, 2022; Paul, 2022). Three enzymes are involved in H_2_S catabolism: sulfide quinone oxidoreductase (SQR), persulfide dioxygenase (ETHE1), and rhodanese (thiosulfate sulfurtransferase, TST) (Hildebrandt and Grieshaber, 2008). The oxidation pathway begins with SQR, which catalyzes the formation of SQR persulfide (SQR-SSH) at one of its two Cys-SH residues (Jackson et al., 2012). SQR is a flavoprotein bound to the inner mitochondrial membrane. As it oxidizes, a noncovalently bound FAD is reduced, and electrons are transferred to coenzyme Q, contributing to the electron transport chain and ATP production. For this reason, H_2_S is considered the first inorganic substrate for oxidative phosphorylation, similar to succinate (Goubern et al., 2007). SQR has a high catalytic rate, ensuring efficient H_2_S catabolism. The sulfur atom from SQR-SSH is transferred to GSH, forming glutathione persulfide (GSSH), which can then be oxidized to sulfite (SO_3_^2−^) by ETHE1 or transferred to sulfite by TST to form thiosulfate. Sulfite is further oxidized to sulfate by sulfite oxidase (SO), or it can directly accept sulfur from SQR-SSH, producing thiosulfate. Some studies suggest that endogenous reducing compounds, such as DHLA, may also accept sulfur from SQR-SSH (Kabil and Banerjee, 2014). Additionally, GSSH can donate sulfur to proteins, forming protein persulfides. H_2_S can also be oxidized by hemoproteins, where thiosulfate and inorganic polysulfides bind to heme iron. Initially, H_2_S binds to Fe^3+^ in methemoglobin (MetHb), and additional H_2_S molecules combine to form persulfide or polysulfide chains (Vitvitsky et al., 2015). These can be oxidized in the presence of oxygen to thiosulfate, which is then released. This oxidation process is particularly important in erythrocytes, which lack mitochondria. Other proteins, such as catalase, superoxide dismutase (SOD), and SOD2, can also oxidize H_2_S to polysulfides, using oxygen or hydrogen peroxide as electron acceptors (Olson et al., 2017; Olson et al., 2018).

## The signaling pathways modulated by H_2_S

More and more studies have demonstrated that H_2_S exerts regulatory effects by regulating diverse signaling pathways (Tao et al., 2024; Zhu et al., 2024), including modulation of epigenetic modifications, protein expression, activity, and localization, as well as influencing the redox microenvironment and interacting with other gasotransmitters such as NO and CO. H_2_S was also found to impact cellular signaling pathways, such as phosphoinositide 3-kinase (PI3K)/protein kinase B (Akt), mitogen-activated protein kinase (MAPK), and Janus kinase (JAK)/signal transducer and activator of transcription (STAT) (Tao et al., 2024). H_2_S influences gene transcription through epigenetic regulation, particularly by modulating DNA methylation, a process involving the addition of methyl groups to DNA that often silences gene expression without altering the nucleotide sequence (Tao et al., 2024). In mammalian cells, DNA methylation predominantly occurs at the C5 position of CpG dinucleotides, facilitated by DNA methyltransferases (DNMTs) (Yang et al., 2025). H_2_S suppresses Dnmt3a transcription via S-sulfhydration of the interferon regulatory factor 1 (IRF1), reducing methylation of the mitochondrial transcription factor A (TFAM) promoter in the vascular system (Li and Yang, 2015). This regulation helps maintain mitochondrial DNA (mtDNA) copy number and supports transcription of mtDNA-encoded genes (Li and Yang, 2015). Histones are proteins that package DNA into stable structural units, and their modification plays a crucial role in regulating gene expression and cellular functions. As epigenetic marks, histone modifications, such as acetylation, methylation, phosphorylation, and ubiquitination, can be inherited and are essential for recruiting or activating downstream effectors (Liu and Si, 2024). H_2_S can enhance the expression and activity of histone deacetylase (HDAC), specifically silent mating type information regulator 2 homolog 1 (SIRT1). This effect is attributed to the S-sulfhydration of SIRT1 by H_2_S, which increases its zinc binding, thereby promoting its stability and deacetylation activity (Calabrese and Scuto, 2020). Activation of SIRT1 by H_2_S protects against diabetic nephropathy (Ahmed et al., 2019), mitigates insulin resistance (IR)-induced apoptosis in cardiomyocytes (Liu et al., 2017), and reduces atherosclerotic plaque formation (Du et al., 2019). Noncoding RNAs, which do not code for proteins, play essential regulatory roles in cellular processes. These RNAs are primarily classified into microRNAs (miRNAs), intronic RNAs, long noncoding RNAs (lncRNAs), circular RNAs, and extracellular RNAs (Sun et al., 2016; Zhang and Sun, 2020b; Zhang and Sun, 2020a; 2021). Several noncoding RNAs have been implicated in the protective effects of H_2_S. For example, upregulation of miR-221 has been shown to attenuate ischemia-induced heart failure (Su et al., 2015; Verjans et al., 2018; Li M. et al., 2020). However, downregulation of miR-21 by the H_2_S donor DATS promotes neovasculogenesis and may prevent cardiovascular diseases (Chiang et al., 2013). Transcription factors (TFs) are proteins that bind to specific DNA sequences to regulate gene transcription. Nuclear factor E2-related factor 2 (Nrf2) is a key TF involved in controlling the expression of numerous antioxidant genes. H_2_S activates Nrf2 by S-sulfhydrating Kelch-like ECH-associated protein 1 (Keap1), which releases Nrf2 from repression, thereby mitigating oxidative stress (Hourihan et al., 2013). Additionally, sulfhydration of Keap1, an E3 ubiquitin ligase substrate adaptor, inhibits its ability to degrade Nrf2, leading to the restoration of antioxidant gene expression. Specific S-sulfhydration sites on Keap1, such as Cys-151 in cellular aging and Cys-151 and Cys-273 in atherosclerosis associated with diabetes, are critical for this antioxidant defense mechanism (Xie et al., 2016). Ubiquitin is first activated by the ubiquitin-activating enzyme (E1), then transferred to the ubiquitin-conjugating enzyme (E2), and finally attached to the substrate by the ubiquitin ligase (E3) with the assistance of substrate adaptors. The ubiquitin-tagged proteins are subsequently recognized and degraded by the proteasome. H_2_S has been shown to reduce the levels of muscle RING finger 1 (MuRF1) and atrogin-1, two muscle-specific E3 ligases, thus mitigating immobilization-induced skeletal muscle atrophy (Lu et al., 2020; Yang et al., 2024). Additionally, H_2_S enhances S-sulfhydration at Cys-44 in MuRF1, which helps prevent cardiac muscle degradation in diabetic cardiomyopathy (DCM) (Sun et al., 2020; Peng et al., 2022). The activation of the PI3K/Akt signaling pathway mediates many protective effects of H_2_S, including the regulation of autophagy, apoptosis, and inflammation. For instance, H_2_S prevents osteoblast apoptosis to alleviate osteoporosis progression (Yan et al., 2017) and reduces reactive oxygen species (ROS) production and inflammation, protecting against ventilator-induced lung injury (Spassov et al., 2017) by activating the PI3K/Akt signaling pathway. In addition to its role in anti-inflammatory and anti-apoptotic processes, H_2_S activates PI3K/Akt signaling in the treatment of neurological diseases (Xu et al., 2018; Li et al., 2021). In addition, H_2_S inhibits MAPK signaling, providing therapeutic benefits in inflammatory and fibrotic conditions. This action protects alveolar epithelial cells against inflammation, injury, and apoptosis via the repression of prolyl hydroxylase 2/HIF-1α/MAPK signaling, suggesting its potential in treating COPD and pulmonary fibrosis (Guan et al., 2020). H_2_S also modulates the mTOR signaling pathway, a key regulator of cellular and organismal metabolism in response to environmental changes (Hou et al., 2017). In high-fat diet (HFD)-induced obesity, H_2_S reverses abnormal activation of mTOR and IKK/NF-κB pathways, alleviating hypothalamic inflammation (Zhao et al., 2022). Furthermore, H_2_S suppresses the JAK/STAT signaling pathway to reduce inflammation and apoptosis, as observed in diabetes-induced myocardial fibrosis (Liu et al., 2018). The downregulation of JAK/STAT and NF-κB signaling by H_2_S also helps recover the protective effects of preconditioning against ischemia-reperfusion injury in aging hearts by reducing ROS levels (Li et al., 2016). In summary, H_2_S plays a crucial role as a gasotransmitter in regulating multiple signaling pathways, contributing to cellular homeostasis and protection against pathological conditions.

## Immune regulatory mechanism of PH

**FIGURE 3:**
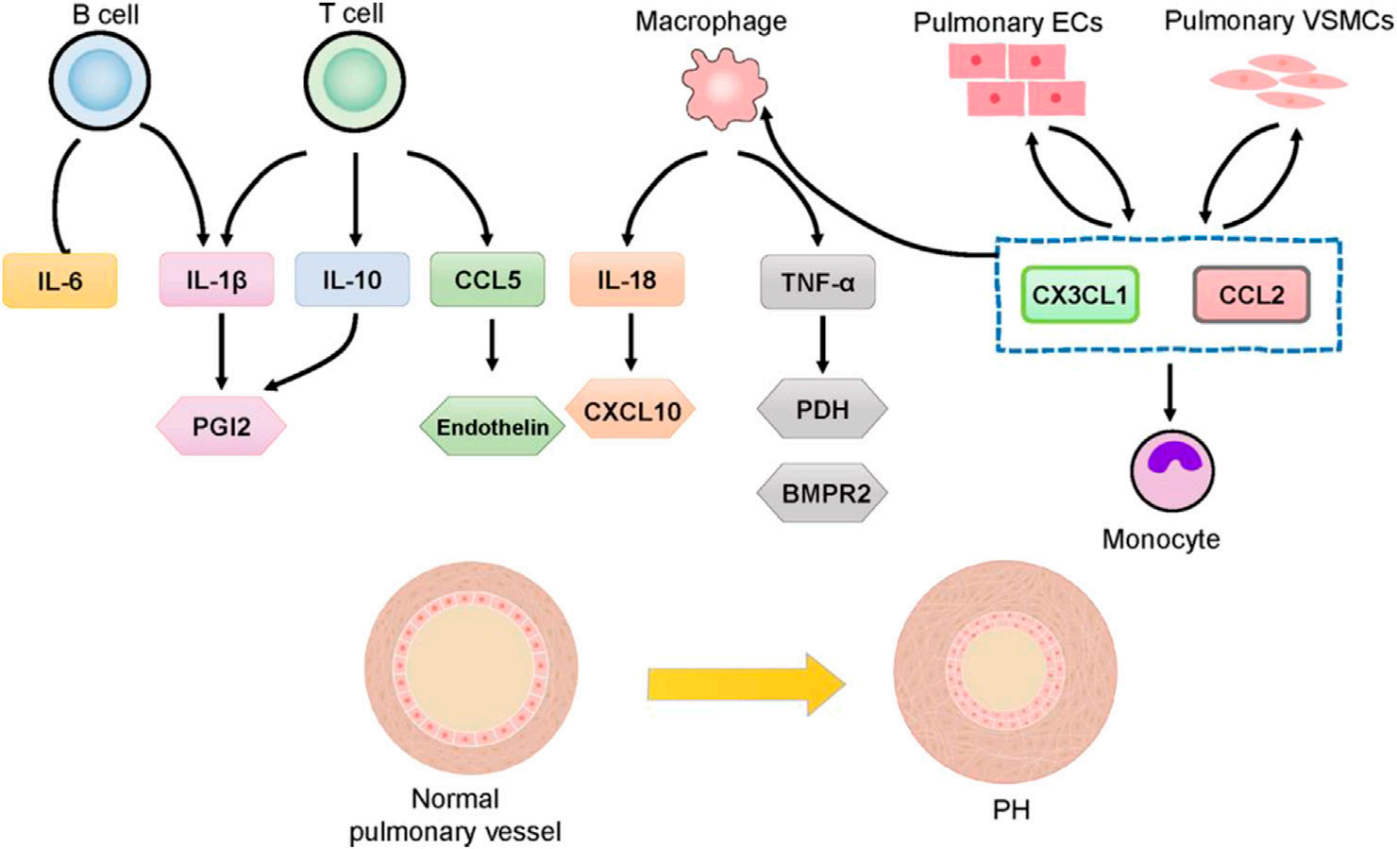
Involvement of cytokines and chemokines in PH.

Pulmonary vascular remodeling in PAH is often marked by perivascular inflammatory infiltration, involving T Cells, B Cells, macrophages, dendritic cells (DCs), mast cells, and neutrophils (Figure 3). This suggests that immune cells play a significant role in pulmonary vascular remodeling (Galiè et al., 2009; Amaya-Uribe et al., 2019). T Cells, particularly helper T Cells (Th cells) and regulatory T Cells (Tregs), are critical components of the adaptive immune response and are pivotal in the pathogenesis of PH (Rabinovitch et al., 2014; Wang R. R. et al., 2022). Th cells promote a pro-inflammatory response, while Tregs maintain self-tolerance and prevent autoimmunity. The balance and homeostasis of T Cells and their cytokines are essential to prevent the loss of self-tolerance, influencing inflammation and PAH progression (Gaowa and Zhou, 2014). Macrophages, key players in the innate immune system, present antigens to T Cells, thereby activating the adaptive immune system (Zhao et al., 2024). Pulmonary inflammation mediated by macrophages is crucial in pulmonary vascular remodeling (El Chami and Hassoun, 2012). In PAH models and patients with left heart disease-related PH, macrophages are elevated, along with increased lung IL-6 levels (Kherbeck et al., 2013). In a chronic thromboembolic PH mouse model, F4/80 positive monocytes/macrophages accumulate in high-flow arteries (Breitling et al., 2017). Increased monocyte recruitment chemokines and peripheral blood monocytes are observed in PH, with monocytes potentially differentiating into perivascular macrophages upon migrating to the pulmonary vasculature (Florentin et al., 2018). This process is facilitated by the activation of chemokines such as chemokine ligand 2 (CCL2) and C-X3-C motif chemokine ligand 1 (CX3CL1) (Florentin et al., 2018). B Cells can differentiate into plasma cells, which produce autoantibodies, and they play a crucial role in immune responses by collaborating with antigen-presenting DCs and lymphoid organs through antigen presentation, cytokine production, and the facilitation of T effector cell differentiation (Kherbeck et al., 2013). B Cells have been shown to play a functional role in PH, with B Cell depletion therapy demonstrating potential in reducing right ventricular systolic pressure and vascular remodeling in experimental PH (Breitling et al., 2017). This therapy has also shown promise as an effective and safe adjuvant treatment for systemic sclerosis-PAH and systemic lupus erythematosus-PAH (Hennigan et al., 2008; Zamanian et al., 2021). The accumulation of perivascular immune cells and intravascular infiltration, coupled with elevated cytokine levels, leads to increased vascular inflammation and dysfunction, playing critical regulatory roles in PAH and correlating with disease severity (Groth et al., 2014). Circulating cytokine levels are important markers for the diagnosis and treatment of PAH, and targeting specific cytokine responses and pathways is considered a promising therapeutic strategy (Groth et al., 2014). Certain cytokines and chemokines have been associated with poor clinical outcomes in PAH patients and may serve as biomarkers for disease progression. For instance, IL-1β and TNF-α are linked to the accumulation of extracellular matrix proteins, such as fibronectin, which are prevalent in PAH lesions. In contrast, IL-6 is associated with the proliferation of VSMCs (Fujita et al., 2002; Zhong et al., 2021). A deeper understanding of the roles of immune cells, cytokines, and chemokines in PH and their impact on abnormal angiogenesis and pulmonary artery remodeling is necessary. Although the immune/inflammatory components of PH require further clarification, recent studies in animal models have shown that anti-inflammatory therapies can reduce or even reverse PH effects. Current therapeutic drugs for PH primarily target vasodilation but also exhibit immunomodulatory properties (Cohen-Kaminsky et al., 2014). However, no approved treatments specifically target the inflammatory processes associated with pulmonary vascular diseases.

Tacrolimus, a calcineurin inhibitor with immunoregulatory and anti-inflammatory properties, has been shown to reverse disease progression in PAH rat models induced by MCT and hypoxia, restore pulmonary artery endothelial cell function, and activate BMPR2 receptor signaling by displacing FKBP12 from the BMPR1 co-receptor (Spiekerkoetter et al., 2013). B Cell depletion therapy using rituximab, an anti-CD20 monoclonal antibody, has reduced rheumatoid factor, IL-12, and IL-17 levels in patients. Statins, known for their anti-inflammatory and immunomodulatory functions, have improved right ventricular remodeling in patients with idiopathic PH, hereditary PH, and connective tissue disease-associated PH (Kao, 2005). Elevated circulating inflammatory markers are observed in patients with chronic embolic PH and PAH, with some patients showing clinical improvement following treatment with corticosteroids or immunosuppressants (Sanchez et al., 2006). The evidence suggests that suppressing immunity and inflammation may be a potential strategy for PH treatment. However, while immunosuppressants effectively inhibit immune cell function and reduce inflammation, no immunosuppressants have been approved for PH treatment, and the therapeutic effects of most immunomodulators are still under clinical investigation.

## Role and mechanisms of H_2_S in HPH

HPH is a critical process that can develop in various cardiac and pulmonary diseases following acute or chronic hypoxia. It is characterized by elevated PAP and increased vascular resistance, resulting from a combination of hypoxic vasoconstriction and vascular remodeling. The pathophysiology of HPH involves progressive pulmonary vasoconstriction, vascular inflammation, oxidative stress, and structural remodeling of pulmonary vessels (Tian, 2008; Yan et al., 2008; Olson et al., 2010; Evans et al., 2011). In early 2003, Tang’s group demonstrated the significance of H_2_S in pulmonary circulation, showing reduced H_2_S levels in lung tissues and plasma, along with inhibited expression and activity of cystathionine γ-lyase (CSE) in the pulmonary arteries and lung tissues of HPH rats (Chunyu et al., 2003). Notably, supplementation with a H_2_S donor significantly reduced PAP and mitigated pulmonary vascular remodeling (Chunyu et al., 2003). Subsequent studies have further elucidated that endogenous H_2_S inhibits the development of HPH, with downregulation of the H_2_S pathway identified as a key mechanism in its progression (Chunyu et al., 2003). Therefore, insufficient H_2_S production contributes to the pathogenesis of HPH (Chen and Wang, 2012; Liu et al., 2012; Yang et al., 2012; Chen et al., 2020; Roubenne and Marthan, 2021).

It is noted that the vasorelaxant mechanisms of H_2_S are relevant for the management of PH (Munteanu et al., 2024). To date, H_2_S exerts vasodilatory effects primarily by activating ATP-sensitive potassium (KATP) channels, causing membrane hyperpolarization and relaxation of VSMCs (Song et al., 2023). Additionally, H_2_S inhibits L-type calcium channels, reducing calcium influx and promoting VSMC relaxation. Beyond ion channel modulation, H_2_S stimulates sGC, increasing cyclic guanosine monophosphate (cGMP) levels, which activate protein kinase G (PKG) (Huerta de la Cruz et al., 2023). PKG then phosphorylates vasodilator-stimulated phosphoprotein (VASP), facilitating smooth muscle relaxation. H_2_S also enhances NO signaling by increasing NO bioavailability through the inhibition of its degradation and activation of endothelial nitric oxide synthase (eNOS), further elevating cGMP levels and enhancing vasodilation (Cirino and Szabo, 2023). In addition, H_2_S modulates endothelium-derived hyperpolarizing factor (EDHF) pathways, promoting VSMC relaxation through gap junctions. H_2_S also activates transient receptor potential (TRP) channels, particularly TRPV4, in endothelial cells, which stimulates calcium entry and the production of vasodilators like NO and prostacyclin, helping regulate vascular tone (Teoh et al., 2020; Liu X. Y. et al., 2022). These multifaceted mechanisms of H_2_S-induced vasodilation highlight its potential for treating vascular diseases such as PH (Lv et al., 2021). The complex interplay between ion channel modulation, cyclic nucleotide signaling, NO pathways, and protein sulfhydration makes H_2_S a promising therapeutic target for managing PH-induced vascular tone dysfunction. H_2_S modulates hypoxia-induced pulmonary vasoconstriction by mitigating ROS production and regulating calcium signaling (Madden et al., 2012). Interestingly, studies on the effects of H_2_S on pulmonary vasodilation function and PH are limited. In spite of this, the ability of H_2_S to counteract endothelial dysfunction and restore vascular homeostasis suggests a promising therapeutic role in managing pulmonary vascular disorders, including PH.

In a rat model of PH established by exposure to normobaric hypoxic conditions for 3 weeks, the H_2_S-generating enzyme activity in lung tissue and pulmonary artery was significantly downregulated (Zhang et al., 2003; Geng et al., 2004). Propargylglycine (PPG), an inhibitor of endogenous H_2_S production worsened PH in hypoxic rats by increasing endogenous production of NO and the expression of NOS in pulmonary arteries (Zhang et al., 2004). This finding indicates that a negative feedback interaction exists between the NO/NOS system and the H_2_S/CSE system in the development of HPH (Zhang et al., 2004). Plasma CO levels and the expression of heme oxygenase (HO-1) were significantly elevated in HPH rats, whereas these upregulations were further aggravated by exogenously applied H_2_S (Qingyou et al., 2004). By contrast, exogenous administration of PPG, an inhibitor of CSE, inhibited the plasma H_2_S contents and worsened HPH by downregulating plasma CO levels and suppressing the expressions of HO-1 in pulmonary arteries (Qingyou et al., 2004), indicating that the crosstalk between H_2_S and CO may participate in the development of HPH. These interconnected gasotransmitter pathways may mutually influence each other, thereby playing a significant regulatory role in the progression of HPH (Zhang et al., 2004).

Oxidative stress plays a role in the development of HPH by regulating the proliferation and migration of pulmonary VSMCs and ECs. Administration of NaHS reduced mean pulmonary artery pressure in HPH rats by reducing the levels of oxidized GSH and enhancing total antioxidant capacity in the lung (Wei et al., 2008). This finding suggests that H_2_S acts as an antioxidant in HPH, partially through attenuating GSSG levels (Wei et al., 2008). Compared with normal rats, the apoptosis of arteriae pulmonalis in HPH rats was significantly lower, as evidenced by increased Bcl-2 protein and decreased Bax protein (Xu et al., 2011). Plasma H_2_S levels and the CSE mRNA level in HPH rats were significantly lower than those in control rats (Xu et al., 2011). These results obtained suggested that the endogenous H_2_S system may be closely linked to HPH, and suppression of the H_2_S/CSE system may increase the Bcl-2/Bax ratio, inhibit apoptosis of pulmonary VSMCs, and ultimately contribute to HPH development (Xu et al., 2011). Prostacyclin (PGI2), an effective but costly vasodilator, is commonly used in the treatment of PH. Endogenous PGI2 formation primarily depends on cyclooxygenase-2 (COX-2) expression, which is reportedly downregulated in hypoxia-induced PAH. H_2_S has been shown to upregulate COX-2 (Hu et al., 2008), but plasma H_2_S levels are reduced during hypoxia-induced pulmonary vascular remodeling (Tian, 2014). It is possible that H_2_S may inhibit hypoxia-induced proliferation of pulmonary VSMCs and mitigate PH by upregulating the COX-2/PGI2 signaling pathway. To explore this, Li et al. assessed the effects of H_2_S on chemical hypoxia-induced proliferation of pulmonary VSMCs (Li et al., 2014). Exposure to cobalt chloride (CoCl_2_) significantly suppressed the COX-2 expression and PGI2 release in pulmonary VSMCs, which was reversed by supplementation of H_2_S (Li et al., 2014). These findings suggest that modulating endogenous H_2_S production or administering H_2_S donors like NaHS could offer a novel therapeutic strategy for PAH by upregulation of the COX-2/PGI2 axis (Li et al., 2014).

H_2_S exerts anti-inflammatory, antioxidative, and other biological effects across various organs. The exaggerated proliferation and apoptosis resistance of pulmonary VSMCs are central to vascular remodeling in PH. It was found that H_2_S significantly inhibited hypoxia-induced pulmonary VSMCs and reversed chronic hypoxia-induced PH by suppressing endoplasmic reticulum (ER) stress markers in both *in vivo* and *in vitro* models (Wu et al., 2019). Also, H_2_S was reported to inhibit activating transcription factor 6 (ATF6) and mitigate the hypoxia-induced decline in mitochondrial calcium levels (Wu et al., 2019). Consequently, H_2_S effectively inhibits hypoxia-induced proliferation, migration, and oxidative stress of pulmonary VSMCs. Attenuating ER stress with exogenous H_2_S represents a promising therapeutic strategy for PH with high translational potential (Wu et al., 2019).

Mesenchymal stem cells (MSCs) derived from the placenta, bone marrow, adipose, and other tissues have emerged as a promising regenerative therapy for PH over the last decade (Pullamsetti et al., 2014). MSC delivery significantly inhibits pulmonary artery remodeling, slowing PAH progression (Malliaras and Marbán, 2011). However, low cell retention and engraftment after delivery pose significant challenges to the effectiveness of MSC-based therapy (Malliaras and Marbán, 2011). Increasing evidence suggests that programmed cell death (PCD), including ferroptosis, is closely linked to the low engraftment and survival rates of transplanted MSCs (Li X. et al., 2020). Ferroptosis, which can be induced in MSCs by erastin, has been shown to affect MSC viability and differentiation (Lan et al., 2022; Han et al., 2024). Inhibiting ferroptosis has been found to maintain MSC viability, enhance differentiation, and improve the efficacy of transplanted MSCs in various disease models (Liu J. et al., 2022). However, the direct impact of ferroptosis on MSC delivery in PAH remains underexplored. *In vivo*, CSE overexpression improved the survival of erastin-treated MSCs delivered to mice with HPH (Hu et al., 2023). *In vitro*, CSE overexpression enhanced H_2_S production and inhibited ferroptosis-related markers in erastin-treated MSCs (Hu et al., 2023). Notably, upregulation of the CSE/H_2_S pathway induced Keap1 S-sulfhydration, which contributed to the inhibition of ferroptosis (Hu et al., 2023). Thereafter, upregulation of the CSE/H_2_S pathway in CMSCs inhibits ferroptosis and enhances their suppressive effect on vascular remodeling in mice with HPH (Hu et al., 2023). The protective effect of the CSE/H_2_S pathway against ferroptosis in CMSCs is mediated via S-sulfhydration of Keap1 and subsequent activation of Nrf2 signaling. This study provides a novel therapeutic avenue for improving the efficacy of transplanted MSCs in PH (Hu et al., 2023). Additionally, the ability of CSE to inhibit ferroptosis suggests that genetic approaches to manipulate CSE expression and H_2_S production could further enhance the protective capacity of MSCs in PH (Hu et al., 2023).

## Role of H_2_S in MCT-induced PH

MCT, a toxic alkaloid, induces the proliferation of pulmonary VSMCs and inflammation of endothelial cells, leading to right heart dysfunction due to cardiac overload. The MCT-induced PH model is widely used as a classical animal model of PH. Evidence for the role of H_2_S in MCT-induced PH is emerging. Previously, Basar et al. found that plasma H_2_S levels were not changed in the MCT-induced PH of rats (Sirmagul et al., 2013). Importantly, they did not examine the direct role of H_2_S in MCT-induced PH (Sirmagul et al., 2013). Later, Jin’s group showed that the endogenous H_2_S/CSE pathway was suppressed in MCT-induced PH rats, and exogenous H_2_S reduced pulmonary artery pressure, reversed pulmonary vascular structural remolding by suppressing phosphorylation of NF-κB p65 and IκBα MCT-treated rats (Feng et al., 2017). Hence, they showed the first evidence that the downregulated H_2_S/CSE pathway was responsible for PH in MCT-induced rat by suppressing NF-κB-mediated endothelial inflammation (Feng et al., 2017). In line with this, Sevin’s group demonstrated that H_2_S levels were reduced, and L-cysteine-induced relaxations were impaired in the pulmonary arteries from MCT-induced PH rats (Turhan et al., 2022). The H_2_S donor, Na_2_S, prevented increased pulmonary artery pressure and hypertrophy by improving endothelial dysfunction (Turhan et al., 2022). Further studies have elucidated the mechanisms by which H_2_S exerts its effects in MCT-induced PH rats (Zhang D. et al., 2019). H_2_S was found to inhibit pulmonary arterial endothelial cell inflammation and prevent vascular remodeling, likely by suppressing the NF-κB signaling pathway and endothelial-mesenchymal transition (EndMT) (Zhang D. et al., 2019; Zhang H. et al., 2019). *In vivo* and *in vitro* findings revealed that H_2_S directly deactivates the inhibitor of kappa B kinase subunit beta (IKKβ) by sulfhydrating its Cys179, thereby preventing NF-κB activation and reducing endothelial cell inflammation in PH (Zhang D. et al., 2019). Additionally, H_2_S was shown to control MCT-induced PH by inhibiting mast cell aggregation and degranulation, as well as the release of interleukin-6 (IL-6) (Zhang D. et al., 2019). The inhibition of the NF-κB pathway and subsequent EndMT in pulmonary arteries further underscores the beneficial effects of H_2_S in the development of MCT-induced PH (Zhang D. et al., 2019).

Interestingly, enhanced endogenous sulfur dioxide (SO_2_) level in endothelial cells and increased enzymatic activity of aspartate aminotransferase (AAT), a major SO_2_ synthesis enzyme, were observed in a rat model of MCH-induced PH (Zhang D. et al., 2018). The H_2_S donor reversed the CSE knockdown-induced increase in endogenous SO_2_ levels and AAT activity (Zhang D. et al., 2018). Mechanistically, H_2_S sulfhydrated AAT1/2 proteins, restoring the reduced sulfhydration of AAT1/2 observed in CSE knockdown endothelial cells (Zhang D. et al., 2018). Furthermore, the AAT inhibitor l-aspartate-β-hydroxamate (HDX), which blocked the CSE knockdown-induced upregulation of the endogenous SO2/AAT pathway, exacerbated the activation of the nuclear factor-κB pathway and increased levels of downstream inflammatory factors, including ICAM-1, TNF-α, and IL-6 in endothelial cells (Zhang D. et al., 2018). *In vivo*, H_2_S restored the MCT-induced deficiency in endogenous H_2_S production and reversed the upregulation of the endogenous SO_2_/AAT pathway via the sulfhydration of AAT1 and AAT2 (Zhang D. et al., 2018). In conclusion, this study demonstrated that H_2_S inhibits endogenous SO_2_ generation by inactivating AAT through the sulfhydration of AAT1/2 (Zhang D. et al., 2018). The increase in endogenous SO_2_ may serve a compensatory role when the H_2_S/CSE pathway is downregulated in MCT-induced PH, thereby providing protective effects against endothelial inflammatory responses (Zhang D. et al., 2018). In summary, H_2_S shows significant potential in mitigating MCT-induced PH by attenuating the activation of the NF-κB pathway, thereby diminishing inflammation in pulmonary arterial endothelial cells and alleviating vascular remodeling. Additionally, H_2_S might protect endothelial cells and other critical immune cells from damage by inhibiting PCD pathways such as ferroptosis, which deserved further studies. H_2_S, as an important immunoregulatory molecule, exhibits significant therapeutic potential in MCT-induced PH. In the future, strategies to regulate H_2_S production or utilize H_2_S donors could offer novel therapeutic approaches, particularly in targeting immunoregulation in MCT-induced PH.

## Role of H_2_S in high pulmonary blood flow-induced PH

PH is a prevalent complication of CHD patients, which is characterized by a left-to-right shunt and elevated pulmonary blood flow. A significant pathological consequence of PH is the structural remodeling of the pulmonary vasculature. The mechanisms underlying the structural changes in response to high pulmonary blood flow remain inadequately understood. Studies have shown that high pulmonary blood flow induces high shear stress on endothelial cells and VSMCs in pulmonary arteries, leading to maladaptive changes in vascular structure and function. In rat models, high pulmonary blood flow-induced PH was established through the creation of an abdominal aorta/inferior vena cava shunt. It was reported that following 4 weeks of shunting, the H_2_S/CSE pathway in lung tissue was upregulated (Xiaohui et al., 2005; Li et al., 2006a). However, after 11 weeks, the pathway was downregulated, coinciding with a marked increase in pulmonary arterial systolic pressure (PASP) and the onset of pulmonary vascular remodeling (Xiaohui et al., 2005; Li et al., 2006a). Supplementation with an exogenous H_2_S donor in shunt rats reduced vascular remodeling and successfully lowered PASP (Shi et al., 2003; Xiaohui et al., 2005; Li et al., 2006a). H_2_S might exert its regulatory effects on PH induced by increased pulmonary blood flow through multiple mechanisms. H_2_S inhibited the proliferation of pulmonary VSMCs via inhibiting the mitogen-activated protein kinase/extracellular signal-regulated kinase (MAPK/ERK) pathway, thereby alleviating pulmonary vascular remodeling in PH rats induced by high pulmonary blood flow (Li et al., 2006a). Additionally, H_2_S suppressed the inflammatory response in pulmonary arteries by downregulating the NF-κB pathway in PH rats with increased pulmonary blood flow (Jin et al., 2008). Several studies revealed that H_2_S promoted collagen degradation within pulmonary artery walls and reduced extracellular matrix (ECM) accumulation, mitigating vascular remodeling and PH induced by high pulmonary blood flow (Li et al., 2008; Lv et al., 2021).

The contents of ICAM-1, IL-8 and MCP-1 contents in lung tissue were higher in shunt rats, this was prevented by H_2_S (Jin et al., 2008). The anti-inflammatory role of H_2_S in PH induced by high pulmonary blood flow was associated with upregulation of IκBα expression and downregulation of NF-κB p65 expression, thus inhibiting the expression of inflammatory related factors (Jin et al., 2008). Of note, H_2_S also modulated the production of vasoactive peptides, including endothelin-1 (ET-1), atrial natriuretic peptide (ANP), calcitonin gene-related peptide (CGRP), and pro-adrenomedullin peptide (PAMP), thereby influencing pulmonary hemodynamics and vascular structure (Li et al., 2006b). Specifically, H_2_S inhibited the production of endogenous vasoconstrictors such as ET-1, ANP, and CGRP, while promoting plasma PAMP levels, leading to vasodilation and relief of PH (Li et al., 2006b). Moreover, H_2_S induced the apoptosis of pulmonary VSMCs by activating the Fas/caspase3 pathway and inhibiting the Bcl-2 pathway, thereby ameliorating high pulmonary blood flow-induced PH (Li et al., 2009). L-arginine is a semi-essential amino acid that serves as a precursor to NO, a critical signaling molecule involved in vascular homeostasis. In the context of PH, L-arginine plays a significant role due to its involvement in NO production through the action of NOS enzymes. NO is a potent vasodilator that helps regulate pulmonary vascular tone, inhibits smooth muscle cell proliferation, and reduces platelet aggregation. It has been revealed that L-arginine ameliorates PH and pulmonary vascular structural remodeling induced by high pulmonary blood flow (Yanfei et al., 2006). L-arginine-mediated attenuation of PH is attributed to the upregulation of the H_2_S/CSE system (Yanfei et al., 2006). This finding suggests that the interaction between the H_2_S and NO pathways is implicated in high pulmonary blood flow-induced pulmonary vascular remodeling and PH (Yanfei et al., 2006). To sum up, H_2_S may show down the development of high pulmonary blood flow-induced PH by inhibiting the proliferation of pulmonary VSMCs, promoting collagen degradation, suppressing the inflammatory response, influencing pulmonary hemodynamics and vascular structure, activating the Fas/caspase3 pathway, inhibiting the Bcl-2 pathway, and interacting with NO. However, the effectiveness of H_2_S supplementation in high pulmonary blood flow-induced PH remains a subject of ongoing research, as its benefits may vary depending on the underlying etiology of the condition and the stage of disease progression. More importantly, the role of H_2_S in modulating immune cell functions within the context of high pulmonary blood flow-induced PH is an emerging area of research with significant therapeutic implications. As a gasotransmitter, H_2_S is known to exert anti-inflammatory, antioxidant, and vasodilatory effects, which are particularly relevant in the pathogenesis of high pulmonary blood flow-induced PH, a condition characterized by vascular remodeling, immune cell infiltration, and elevated pulmonary pressures. Targeting the H_2_S pathway in immune cells may offer a novel strategy to attenuate the inflammatory and remodeling processes in high pulmonary blood flow-induced PH, potentially improving clinical outcomes in patients with this challenging condition. Future research should focus on elucidating the specific immune cell populations affected by H_2_S, the molecular pathways involved, and the potential for combination therapies that enhance H_2_S signaling in conjunction with other treatments for high pulmonary blood flow-induced PH.

## Role of H_2_S in PH associated with COPD

PH is a common and serious complication of COPD. The relationship between PH and COPD is multifaceted, with PH contributing to the morbidity and mortality associated with COPD. In COPD, chronic inflammation, alveolar destruction, and hypoxia lead to structural and functional changes in the pulmonary vasculature. The destruction of alveolar walls reduces the number of capillaries in the lungs, increasing vascular resistance and PAP. The development of PH in COPD patients significantly worsens the prognosis. It is associated with increased exertional dyspnea, reduced exercise capacity, and decreased survival. PH can exacerbate right ventricular dysfunction due to the increased workload placed on the right heart to pump blood through the narrowed pulmonary arteries, eventually leading to right heart failure. Understanding the relationship between these conditions is crucial for optimizing the management and improving outcomes in patients with COPD. Further research is needed to better understand the mechanisms driving PH in COPD and to develop targeted therapies that can effectively address this challenging complication.

Serum H_2_S levels were diminished in patients with COPD-related PH and H_2_S levels were negatively correlated with PASP, indicating that H_2_S may play an important role in the pathogenesis of COPD-related PH (Yuan et al., 2019; Liao et al., 2021). Chen et al. found that serum H_2_S levels were significantly higher in patients with stable COPD compared to controls and patients with acute exacerbation of COPD (AECOPD) (Chen et al., 2005). Serum H_2_S levels also varied significantly with the severity of airway obstruction in stable COPD, being lower in patients with stage III obstruction compared to those with stage I obstruction (Chen et al., 2005). Furthermore, AECOPD patients with PH exhibited lower serum H_2_S levels than those with normal resting PASP (Chen et al., 2005). Serum H_2_S levels in COPD patients were positively correlated with NO levels, FEV_1_% predicted values, and the percentages of sputum lymphocytes and macrophages (Chen et al., 2005). Conversely, serum H_2_S was negatively correlated with PASP and the percentage of sputum neutrophils (Chen et al., 2005). These results suggest that endogenous H_2_S may play a role in the pathogenesis of PH associated with COPD and could serve as a noninvasive marker of disease activity and severity (Chen et al., 2005). Han and colleagues investigated the role of H_2_S in COPD pathogenesis using a mouse model of tobacco smoke (TS)-induced emphysema (Han et al., 2011). TS exposure significantly decreased the protein expression of CSE, CBS, nuclear erythroid-related factor 2 (Nrf2), phosphorylated-Akt (P (ser473)-Akt), and the glutathione/oxidized GSH ratio in the lung, effects that were largely reversed by H_2_S (Han et al., 2011). NaHS treatment mitigated TS-induced increases in mean linear intercepts, bronchial wall thickness, and total cell counts in bronchoalveolar lavage fluid, including neutrophils, monocytes, and tumor necrosis factor α (Han et al., 2011). Furthermore, NaHS attenuated the rise in right ventricular systolic pressure, pulmonary vascular wall thickness, and the ratio of RV/LV+S in TS-exposed mice (Han et al., 2011). Notably, Akt knockdown abrogated the protective effects of NaHS against TS-induced apoptosis and the downregulation of Nrf2, CGL, and CBS in pulmonary artery endothelial cells (Han et al., 2011). These findings demonstrate that NaHS confers protection against TS-induced oxidative stress, airway inflammation, and remodeling, thereby attenuating the progression of emphysema and PH in COPD mice (Han et al., 2011). The usage of H_2_S donors may hold therapeutic potential for the prevention and treatment of COPD caused by TS exposure (Han et al., 2011; Chen and Wang, 2012). These results indicated that H_2_S might have clinical significance in the treatment of COPD-related PH by exerting anti-inflammatory and anti-oxidative effects.

## Therapeutic potential of organosulfur compounds in PH

In addition to *A. sativum* derivatives, other natural sulphur compounds show promise for the management of PH (Mirhadi et al., 2024). Organosulfur compounds (OSCs)are subdivided into allylic sulfur compounds, indoles, isothiocyanates and sulforaphane (SFN) which are known for their therapeutic characteristics such as anti-inflammatory, antioxidative and antimicrobial properties (Kim et al., 2018; Putnik et al., 2019). The sulfur atom in OSCs underpins their unique properties, making them effective in treating oxidative stress-mediated diseases. OSCs are known to activate the Nrf2 signaling pathway, a critical therapeutic target for combating oxidative stress (Egbujor and Petrosino, 2022). Additionally, OSCs have demonstrated anti-inflammatory effects in both *in vitro* studies and clinical settings (Sahu, 2002). Glucosinolates (GSTs), precursors of isothiocyanates, are abundant in plants of the *Brassica* (Cruciferous) genus. When chewed or damaged by insects, GSTs undergo hydrolysis by the enzyme myrosinase, producing isothiocyanates, thiocyanates, and other bioactive compounds. In the *Allium* genus, organosulfur compounds (OSCs) are classified into two types: (1) volatile, oil-soluble sulfur compounds, such as diallyl sulfides, responsible for their characteristic pungent aroma and flavor, and (2) less odorous, water-soluble compounds like S-allylmercaptocysteine (SAMC) and S-allylcysteine (SAC), found in aqueous *A. sativum* extracts. The pharmacological activity of *A. sativum*-derived OSCs is primarily due to allicin, which forms in high concentrations when *A. sativum* is crushed or cut (Iciek et al., 2009; Lawson and Hunsaker, 2018). Sulforaphane (SFN), a 4-methylsulfinylbutyl isothiocyanate, is a biologically active phytonutrient primarily derived from the Brassica family, especially broccoli (Mirhadi et al., 2024). Its isothiocyanate group ensures a bioavailability of approximately 80%, with broccoli sprouts containing nearly ten times the SFN concentration of mature broccoli (Jaiswal et al., 2011). SFN exhibits diverse biological properties, including antioxidant, antimicrobial, anti-inflammatory, immunomodulatory, chemopreventive, cardioprotective, and neuroprotective effects (Angeloni et al., 2015; Qi et al., 2016; Romeo and Iori, 2018). In a study by Kang et al. (2020), PH and right ventricular dysfunction were induced in hypoxic male mice using semaxanib (SU5416), a VEGFR inhibitor (Wang Y. et al., 2022). Treatment with SFN improved right ventricular structure and function as assessed by transthoracic echocardiography. SFN upregulated Nrf2 expression and its downstream gene NQO1, reduced the inflammatory mediator NLRP3, and mitigated Su5416 and hypoxia (SuHx)-induced RV remodeling (Wang Y. et al., 2022). It also attenuated pulmonary vascular remodeling, fibrosis, and inflammation (Kang et al., 2020). SFN treatment reversed elevated right ventricular systolic pressure (RVSP) and improved right ventricular diastolic/systolic function in SuHx-induced PAH mice (Zhang et al., 2022). In hypoxic PAH models, SFN enhanced apoptosis in pulmonary artery smooth muscle cells (PASMCs) while reducing apoptosis in pulmonary microvascular endothelial cells (Zhang et al., 2022). It lowered right ventricular hypertrophy and pulmonary artery remodeling, reduced IL-6 and TNF-α levels in the lungs and serum, and inhibited NF-κB activation in PASMCs (Zhang et al., 2022). Additionally, SFN boosted antioxidant defenses by increasing total GSH levels, SOD activity, SOD2 expression, and the GSH/GSSG ratio. This was accompanied by a reduction in ROS production, as well as serum malondialdehyde (MDA) content (Qin and Qiao, 2022; Pan et al., 2023). These effects collectively highlight the potential of SFN to mitigate oxidative stress, inflammation, and structural remodeling in PH. Therefore, the protective effects of sulfur-containing compounds such as sulforaphane are well recognized. These compounds offer a foundation for future research and development of H_2_S-based anti-PH drugs.

## Conclusions and future directions

**FIGURE 4:**
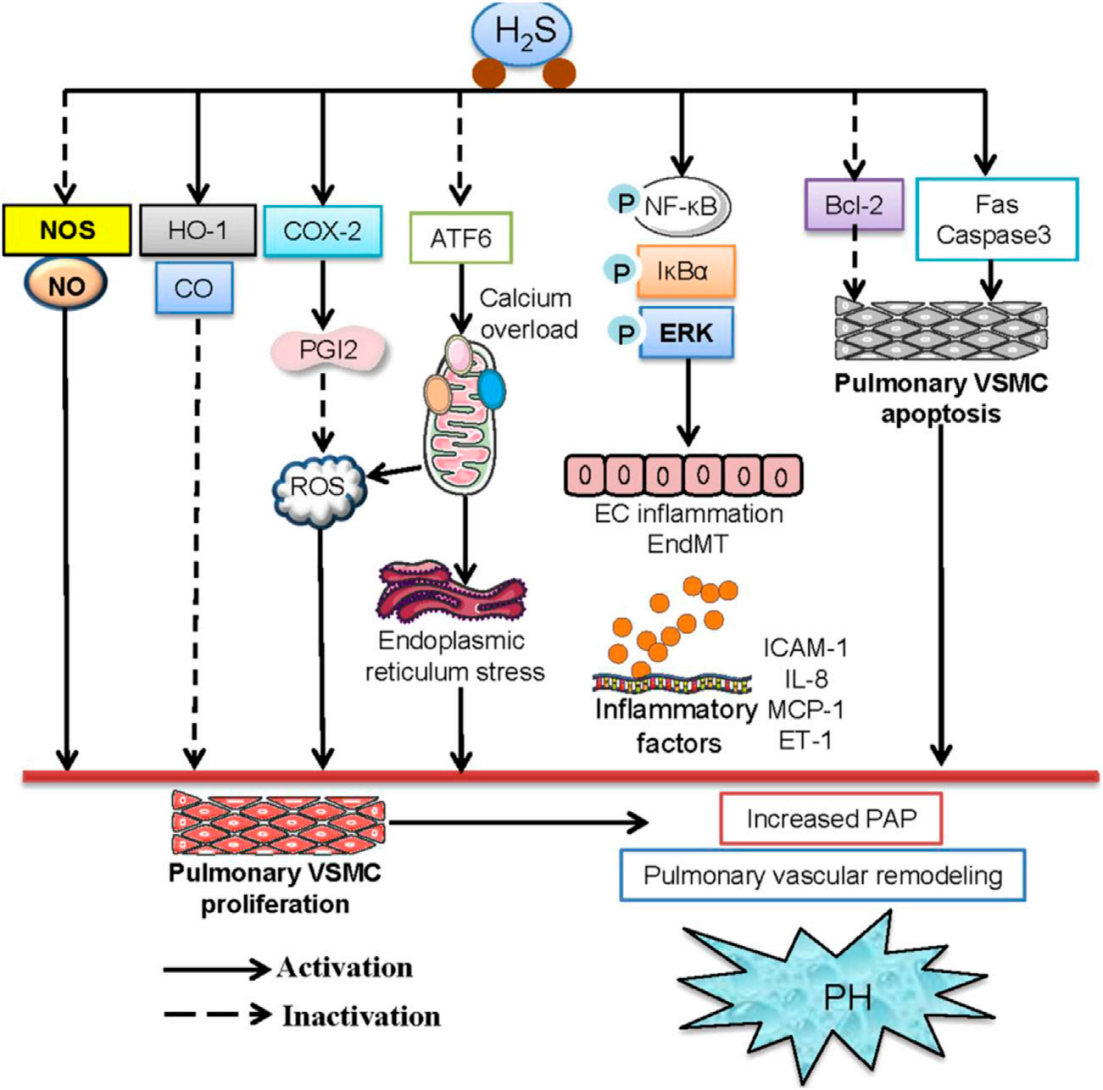
The signaling pathways involved in the protection of H_2_S against PH. H_2_S ameliorated PH by decreasing endogenous production of NO and the expression of NOS and elevating the HO-1/CO pathway in pulmonary arteries. H_2_S may inhibit hypoxia-induced proliferation of pulmonary VSMCs and mitigate PH by upregulating the COX-2/PGI2 signaling pathway. H_2_S was reported to inhibit ATF6 and mitigate the hypoxia-induced decline in mitochondrial calcium levels. H_2_S effectively inhibits hypoxia-induced proliferation, migration, oxidative stress, and ER stress of pulmonary VSMCs. H_2_S reduced pulmonary artery pressure, reversed pulmonary vascular structural remolding by suppressing phosphorylation of NF-κB p65 and IκBα, thus downregulating the levels of downstream inflammatory factors, including ICAM-1, TNF-α, and IL-6 in pulmonary endothelial cells. H_2_S induced the apoptosis of pulmonary VSMCs by activating the Fas/caspase3 pathway and inhibiting the Bcl-2 pathway, thereby ameliorating high pulmonary blood flow-induced PH.

As a recognized vasodilator, H_2_S has prompted investigations into its effects on PH and related diseases such as CHD and COPD. H_2_S inhibits pulmonary VSMC proliferation, modulates vascular cell apoptosis, prevents collagen remodeling, and induces pulmonary vasodilation via KATP channel activation (Figure 4) (Tang et al., 2006; Yang et al., 2008), mitigating vascular endothelial cell inflammation, and regulating immune cell functions. Furthermore, H_2_S interacts with CO and NO signaling pathways to maintain pulmonary vascular function and normal circulation (Roubenne and Marthan, 2021). However, under certain pathological conditions, the endogenous H_2_S is downregulated, contributing to the onset and development of PH. Although the precise cellular mechanisms underlying these dysregulations remain unclear, reduced H_2_S bioavailability is pivotal in promoting endothelial dysfunction, exacerbated inflammation, oxidative stress, and altered smooth muscle cell proliferation within the pulmonary artery walls. Future clinical investigations may consider H_2_S and its related metabolic factors (such as L-cysteine, homocysteine, CSE, CBS, and 3-MST as relevant biomarkers for assessing the prognosis and risk of developing PH from CHD and COPD.

Evidence suggests that H_2_S offers protective effects in PH, positioning it as a potential target for new treatment strategies involving H_2_S-releasing molecules (Table 1). These therapeutic benefits have been primarily demonstrated in studies utilizing H_2_S donors, with NaHS and Na_2_S being the most widely used due to their cost-effectiveness, water solubility, and ability to rapidly release H_2_S under physiological conditions. Sulfide salts, for instance, rapidly release H_2_S, enabling quick and efficient distribution. In healthy individuals, intravascular administration of Na_2_S has been shown to elevate blood concentrations of H_2_S and thiosulfates, as well as exhaled H_2_S levels, within minutes of injection (Toombs et al., 2010). However, the rapid release of H_2_S from sulfide salts can lead to high concentrations that may negatively impact mitochondrial function, particularly in susceptible individuals, such as PH patients. Consequently, the slow-releasing H_2_S donors, such as GYY4137 and dithiolthione compounds, offer an attractive alternative, providing controlled release and sustained H_2_S bioavailability that more closely mimics endogenous production rates, thereby reducing the risk of bolus effects (Giustarini et al., 2010; Rose et al., 2015). The natural H_2_S donors, such as *A. sativum* extracts, have been shown to significantly lower blood pressure in hypertensive patients in multiple clinical trials (Ried, 2016). More evidence supporting the therapeutic potential of H_2_S in treating PH is highly required, and the concerning immunomodulatory mechanisms by which H_2_S influences the pulmonary vascular systems need to be fully elucidated.

**TABLE 1:** The therapeutic impact of H_2_S on the improvement of PH.

H_2_S regulators	Animal or cells	Effects	Mechanisms	References
NaHS	Hypoxic PH rats	Amelioration of PH and pulmonary vascular remodeling	Increased plasma H_2_S level and upregulated CSE in lung tissue	Chunyu et al. (2003)
NaHS	Normobaric hypoxia-induced broilers	Amelioration of PH and right ventricular hypertrophy	Increased plasma concentrations of and H_2_S	Yang et al. (2012)
NaHS	Hypoxic PH rats	Amelioration of PH and pulmonary vascular remodeling	Inhibition of elastin expression of extracellular matrix accumulation	Chen et al. (2020)
Cysteine	Hypoxic rat lung	Reduction in the increases in arterial pressure during hypoxia	Decreased hypoxic pulmonary vasoconstriction	Madden et al. (2012)
NaHS	Hypoxic PH rats	Amelioration of PH and pulmonary vascular remodeling	Increased CSE activity and CSE mRNA in lung tissue	Zhang et al. (2003)
PPG	Hypoxic PH rats	Aggravation of PH	Increased endogenous production of NO	Zhang et al. (2004)
NaHS	Hypoxic PH rats	Amelioration of PH and pulmonary vascular remodeling	Increased plasma CO level and the expressions of HO-1 in pulmonary arteries	Qingyou et al. (2004)
NaHS	Hypoxic PH rats	Amelioration of PH and pulmonary vascular remodeling	Attenuation of oxidized glutathione content	Wei et al. (2008)
NaHS	Hypoxia-induced pulmonary VSMCs	Attenuation of cell proliferation	Upregulation of the COX-2/PGI(2) axis	Li et al. (2014)
GYY4137	Hypoxic PH rats	Decreased pulmonary vascular resistance, and pulmonary artery remodeling	Inactivation of activating transcription factor 6 activation, and decreased mitochondrial calcium	Wu et al. (2019)
GYY4137	Hypoxia-induced pulmonary VSMCs	Attenuation of cell proliferation	Inactivation of activating transcription factor 6 activation, and decreased mitochondrial calcium	Wu et al. (2019)
NaHS	MCT-induced PH rats	Attenuation of PH and pulmonary vascular remolding	Attenuation of endothelial inflammation and NF-κB inactivation	Feng et al. (2017)
NaHS	Hypoxia-induced pulmonary arterial endothelial cells	Attenuation of pulmonary inflammatory response	Attenuation of endothelial inflammation and NF-κB inactivation	Feng et al. (2017)
Na_2_S	MCT-induced PH rats	Attenuation of PH and pulmonary vascular remolding	Promotion of pulmonary relaxation	Turhan et al. (2022)
NaHS	MCT-induced PH rats	Attenuation of PH and pulmonary endothelial inflammation	Activation of IKKβ via sulfhydrating IKKβ at Cys179 to inhibit NF-κB	Zhang D. et al. (2019)
NaHS	MCT-induced PH rats	Attenuation of PH and pulmonary endothelial inflammation	Inhibition of the NF-κB-Snail pathway and the subsequent EndMT in pulmonary arteries	Zhang H. et al. (2019)
NaHS	MCT-induced PH rats	Attenuation of PH and pulmonary vascular remolding	Upregulation of sulfur dioxide (SO_2_)	Zhang D. et al. (2018)
NaHS	High pulmonary blood flow-induced PH rats	Attenuation of PVSR and downregulation of PCNA expression	Inhibition of the ERK/MAPK, NO/NOS pathway and activation of the CO/HO pathway	Li et al. (2006a)
NaHS	High pulmonary blood flow-induced PH rats	Attenuation of PH and pulmonary endothelial inflammation	Inhibition of the NF-κB-Snail pathway	Jin et al. (2008)
NaHS	High pulmonary blood flow-induced PH rats	Attenuation of PH and pulmonary endothelial inflammation	Regulation of vasoactive peptide ET-1,+ ANP, CGRP and PAMP	Li et al. (2006b)
NaHS	High pulmonary blood flow-induced PH rats	Attenuation of PH and pulmonary vascular remolding	Activation of the Fas/caspase3 pathway and inhibition of the Bcl-2 pathway	Li et al. (2009)
NaHS	Tobacco smoke (TS)-induced emphysema mice	Attenuation of mean linear intercepts, bronchial wall thickness	Upregulation of Nrf2, CGL, and CBS in pulmonary artery endothelial cells	Han et al. (2011)
SFN	SU5416 and 10% hypoxia (SuHx)-induced PH mice	Attenuation of pulmonary vascular remodeling, fibrosis, and inflammation	Activation of Nrf2 and inhibition of NLRP3 inflammasome	Kang et al. (2020)
SFN	SU5416 and 10% hypoxia (SuHx)-induced PH mice	Attenuation of right ventricular dysfunction	Activation of Nrf2	Zhang et al. (2022)
SFN	Chronic hypoxia-induced PH mcie	Attenuation of right ventricular hypertrophy and pulmonary arteries remodeling	Inhibition of inflammation and oxidative stress	Pan et al. (2023)
Allicin	MCT-induced PH rats	Attenuation of right ventricular dysfunction	Decrease in medial wall thickness of pulmonary arteries	Bombicz et al. (2017)
Allicin	MCT-induced PH rats	Prevention of pulmonary vessel wall thickness	Inhibition of inflammation and fibrosis	Sánchez-Gloria et al. (2021)
Allicin	MCT-induced PH rats	Attenuation of right ventricular hypertrophy and pulmonary arteries remodeling	Improvement of coronary endothelial function and vasoreactivity	Sun and Ku (2006)

In summary, H_2_S-releasing molecules have shown considerable potential in attenuating vascular and cardiac alterations associated with PH. The development of both synthetic and natural H_2_S-releasing molecules offers a promising avenue for new therapeutic strategies for PH, warranting further preclinical investigations before clinical implementation.

## References

[B1] Ahmed H. H. Taha F. M. Omar H. S. Elwi H. M. Abdelnasser M. (2019). Hydrogen sulfide modulates SIRT1 and suppresses oxidative stress in diabetic nephropathy. Mol. Cell. Biochem. 457, 1–9. 10.1007/s11010-019-03506-x 30778838

[B2] Amaya-Uribe L. Rojas M. Azizi G. Anaya J. M. Gershwin M. E. (2019). Primary immunodeficiency and autoimmunity: a comprehensive review. J. Autoimmun. 99, 52–72. 10.1016/j.jaut.2019.01.011 30795880

[B3] Angeloni C. Malaguti M. Rizzo B. Barbalace M. C. Fabbri D. Hrelia S. (2015). Neuroprotective effect of sulforaphane against methylglyoxal cytotoxicity. Chem. Res. Toxicol. 28, 1234–1245. 10.1021/acs.chemrestox.5b00067 25933243

[B4] Austin E. D. Loyd J. E. (2014). The genetics of pulmonary arterial hypertension. Circ. Res. 115, 189–202. 10.1161/CIRCRESAHA.115.303404 24951767 PMC4137413

[B5] Azaredo Raposo M. Inácio Cazeiro D. Guimarães T. Lousada N. Freitas C. Brito J. (2024). Pulmonary arterial hypertension: navigating the pathways of progress in diagnosis, treatment, and patient care. Rev. Port. Cardiol. 43, 699–719. 10.1016/j.repc.2024.03.004 38972452

[B6] Benavides G. A. Squadrito G. L. Mills R. W. Patel H. D. Isbell T. S. Patel R. P. (2007). Hydrogen sulfide mediates the vasoactivity of garlic. Proc. Natl. Acad. Sci. U. S. A. 104, 17977–17982. 10.1073/pnas.0705710104 17951430 PMC2084282

[B7] Bian J. S. Chen J. Zhang J. Tan J. Chen Y. Yang X. (2024). ErbB3 governs endothelial dysfunction in hypoxia-induced pulmonary hypertension. Circulation 150, 1533–1553. 10.1161/CIRCULATIONAHA.123.067005 38214194

[B8] Bombicz M. Priksz D. Varga B. Kurucz A. Kertész A. Takacs A. (2017). A novel therapeutic approach in the treatment of pulmonary arterial hypertension: Allium ursinum liophylisate alleviates symptoms comparably to Sildenafil. Int. J. Mol. Sci. 18, 1436. 10.3390/ijms18071436 28677661 PMC5535927

[B9] Breitling S. Hui Z. Zabini D. Hu Y. Hoffmann J. Goldenberg N. M. (2017). The mast cell-B cell axis in lung vascular remodeling and pulmonary hypertension. Am. J. Physiol. Lung Cell. Mol. Physiol. 312, L710–l721. 10.1152/ajplung.00311.2016 28235950

[B10] Calabrese V. Scuto M. Salinaro A. T. Dionisio G. Modafferi S. Ontario M. L. (2020). Hydrogen sulfide and carnosine: modulation of oxidative stress and inflammation in kidney and brain Axis. Antioxidants (Basel). 9, 1303. 10.3390/antiox9121303 33353117 PMC7767317

[B11] Castillo-Galán S. Parra V. Cuenca J. (2025). Unraveling the pathogenesis of viral-induced pulmonary arterial hypertension: possible new therapeutic avenues with mesenchymal stromal cells and their derivatives. Biochim. Biophys. Acta Mol. Basis Dis. 1871, 167519. 10.1016/j.bbadis.2024.167519 39332781

[B12] Cerda M. M. Pluth M. D. (2018). S marks the spot: linking the antioxidant activity of N-acetyl cysteine to H(2)S and sulfane sulfur species. Cell. Chem. Biol. 25, 353–355. 10.1016/j.chembiol.2018.04.001 29677486

[B13] Chen J. Zhang H. Yu W. Chen L. Wang Z. Zhang T. (2020). Expression of pulmonary arterial elastin in rats with hypoxic pulmonary hypertension using H2S. J. Recept Signal Transduct. Res. 40, 383–387. 10.1080/10799893.2020.1738482 32160810

[B14] Chen Y. Wang R. (2012). The message in the air: hydrogen sulfide metabolism in chronic respiratory diseases. Respir. Physiol. Neurobiol. 184, 130–138. 10.1016/j.resp.2012.03.009 22476058

[B15] Chen Y. H. Yao W. Z. Geng B. Ding Y. L. Lu M. Zhao M. W. (2005). Endogenous hydrogen sulfide in patients with chronic obstructive pulmonary disease. Zhonghua Jie He He Hu Xi Za Zhi 28, 694–697. 16255955

[B16] Chiang E. P. Chiu S. C. Pai M. H. Wang Y. C. Wang F. Y. Kuo Y. H. (2013). Organosulfur garlic compounds induce neovasculogenesis in human endothelial progenitor cells through a modulation of MicroRNA 221 and the PI3-K/Akt signaling pathways. J. Agric. Food Chem. 61, 4839–4849. 10.1021/jf304951p 23663050

[B17] Chiku T. Padovani D. Zhu W. Singh S. Vitvitsky V. Banerjee R. (2009). H2S biogenesis by human cystathionine gamma-lyase leads to the novel sulfur metabolites lanthionine and homolanthionine and is responsive to the grade of hyperhomocysteinemia. J. Biol. Chem. 284, 11601–11612. 10.1074/jbc.M808026200 19261609 PMC2670165

[B18] Chunyu Z. Junbao D. Dingfang B. Hui Y. Xiuying T. Chaoshu T. (2003). The regulatory effect of hydrogen sulfide on hypoxic pulmonary hypertension in rats. Biochem. Biophys. Res. Commun. 302, 810–816. 10.1016/s0006-291x(03)00256-0 12646242

[B19] Cirino G. Szabo C. Papapetropoulos A. (2023). Physiological roles of hydrogen sulfide in mammalian cells, tissues, and organs. Physiol. Rev. 103, 31–276. 10.1152/physrev.00028.2021 35435014

[B20] Cohen-Kaminsky S. Hautefort A. Price L. Humbert M. Perros F. (2014). Inflammation in pulmonary hypertension: what we know and what we could logically and safely target first. Drug Discov. Today 19, 1251–1256. 10.1016/j.drudis.2014.04.007 24747559

[B21] Cooper C. E. Brown G. C. (2008). The inhibition of mitochondrial cytochrome oxidase by the gases carbon monoxide, nitric oxide, hydrogen cyanide and hydrogen sulfide: chemical mechanism and physiological significance. J. Bioenerg. Biomembr. 40, 533–539. 10.1007/s10863-008-9166-6 18839291

[B22] Devaughn H. Rich H. E. Shadid A. Vaidya P. K. Doursout M. F. Shivshankar P. (2024). Complement immune system in pulmonary hypertension-cooperating roles of circadian rhythmicity in complement-mediated vascular pathology. Int. J. Mol. Sci. 25, 12823. 10.3390/ijms252312823 39684535 PMC11641342

[B23] Doktor F. Antounians L. Figueira R. L. Khalaj K. Duci M. Zani A. (2025). Amniotic fluid stem cell extracellular vesicles as a novel fetal therapy for pulmonary hypoplasia: a review on mechanisms and translational potential. Stem Cells Transl. Med. 14, szae095. 10.1093/stcltm/szae095 39823257 PMC11740888

[B24] Du C. Lin X. Xu W. Zheng F. Cai J. Yang J. (2019). Sulfhydrated sirtuin-1 increasing its deacetylation activity is an essential epigenetics mechanism of anti-atherogenesis by hydrogen sulfide. Antioxid. Redox Signal 30, 184–197. 10.1089/ars.2017.7195 29343087

[B25] Egbujor M. C. Petrosino M. Zuhra K. Saso L. (2022). The role of organosulfur compounds as Nrf2 activators and their antioxidant effects. Antioxidants (Basel). 11, 1255. 10.3390/antiox11071255 35883746 PMC9311638

[B26] El Chami H. Hassoun P. M. (2012). Immune and inflammatory mechanisms in pulmonary arterial hypertension. Prog. Cardiovasc Dis. 55, 218–228. 10.1016/j.pcad.2012.07.006 23009917 PMC3459180

[B27] Evans A. M. Hardie D. G. Peers C. Mahmoud A. (2011). Hypoxic pulmonary vasoconstriction: mechanisms of oxygen-sensing. Curr. Opin. Anaesthesiol. 24, 13–20. 10.1097/ACO.0b013e3283421201 21157304 PMC3154643

[B28] Feng S. Chen S. Yu W. Zhang D. Zhang C. Tang C. (2017). H(2)S inhibits pulmonary arterial endothelial cell inflammation in rats with monocrotaline-induced pulmonary hypertension. Lab. Invest. 97, 268–278. 10.1038/labinvest.2016.129 27941756

[B29] Florentin J. Coppin E. Vasamsetti S. B. Zhao J. Tai Y. Y. Tang Y. (2018). Inflammatory macrophage expansion in pulmonary hypertension depends upon mobilization of blood-borne monocytes. J. Immunol. 200, 3612–3625. 10.4049/jimmunol.1701287 29632145 PMC5940510

[B30] Fujita M. Mason R. J. Cool C. Shannon J. M. Hara N. Fagan K. A. (2002). Pulmonary hypertension in TNF-alpha-overexpressing mice is associated with decreased VEGF gene expression. J. Appl. Physiol. (1985) 93, 2162–2170. 10.1152/japplphysiol.00083.2002 12391106

[B31] Furne J. Saeed A. Levitt M. D. (2008). Whole tissue hydrogen sulfide concentrations are orders of magnitude lower than presently accepted values. Am. J. Physiol. Regul. Integr. Comp. Physiol. 295, R1479–R1485. 10.1152/ajpregu.90566.2008 18799635

[B32] Galiè N. Hoeper M. M. Humbert M. Torbicki A. Vachiery J. L. Barbera J. A. (2009). Guidelines for the diagnosis and treatment of pulmonary hypertension. Eur. Respir. J. 34, 1219–1263. 10.1183/09031936.00139009 19749199

[B33] Gaowa S. Zhou W. Yu L. Zhou X. Liao K. Yang K. (2014). Effect of Th17 and Treg axis disorder on outcomes of pulmonary arterial hypertension in connective tissue diseases. Mediat. Inflamm. 2014, 247372. 10.1155/2014/247372 PMC4158110 25214713

[B34] Ge Q. Zhang T. Yu J. Lu X. Xiao S. Zhang T. (2024). A new perspective on targeting pulmonary arterial hypertension: programmed cell death pathways (Autophagy, Pyroptosis, Ferroptosis). Biomed. Pharmacother. 181, 117706. 10.1016/j.biopha.2024.117706 39581144

[B35] Geng B. Yan H. Zhong G. Z. Zhang C. Y. Chen X. B. Jiang H. F. (2004). Hydrogen sulfide: a novel cardiovascular functional regulatory gas factor. Beijing Da Xue Xue Bao Yi Xue Ban. 36, 106. 14970901

[B36] Gerges C. Matsubara H. Lang I. (2024). Low diffusing capacity for carbon monoxide in chronic thromboembolic pulmonary hypertension: a biomarker for microvascular disease? Heart 110, 1109–1110. 10.1136/heartjnl-2024-324237 39084710 PMC11420748

[B37] Gerő D. Torregrossa R. Perry A. Waters A. Le-Trionnaire S. Whatmore J. L. (2016). The novel mitochondria-targeted hydrogen sulfide (H(2)S) donors AP123 and AP39 protect against hyperglycemic injury in microvascular endothelial cells *in vitro* . Pharmacol. Res. 113, 186–198. 10.1016/j.phrs.2016.08.019 27565382 PMC5113977

[B38] Giustarini D. Del Soldato P. Sparatore A. Rossi R. (2010). Modulation of thiol homeostasis induced by H2S-releasing aspirin. Free Radic. Biol. Med. 48, 1263–1272. 10.1016/j.freeradbiomed.2010.02.014 20171274

[B39] Goubern M. Andriamihaja M. Nübel T. Blachier F. Bouillaud F. (2007). Sulfide, the first inorganic substrate for human cells. Faseb J. 21, 1699–1706. 10.1096/fj.06-7407com 17314140

[B40] Groth A. Vrugt B. Brock M. Speich R. Ulrich S. Huber L. C. (2014). Inflammatory cytokines in pulmonary hypertension. Respir. Res. 15, 47. 10.1186/1465-9921-15-47 24739042 PMC4002553

[B41] Guan R. Wang J. Li D. Li Z. Liu H. Ding M. (2020). Hydrogen sulfide inhibits cigarette smoke-induced inflammation and injury in alveolar epithelial cells by suppressing PHD2/HIF-1α/MAPK signaling pathway. Int. Immunopharmacol. 81, 105979. 10.1016/j.intimp.2019.105979 31771816

[B42] Halimi J. M. Sarafidis P. Azizi M. Bilo G. Burkard T. Bursztyn M. (2024). Screening and management of hypertensive patients with chronic kidney disease referred to Hypertension Excellence Centres among 27 countries. A pilot survey based on questionnaire. J. Hypertens. 42, 1544–1554. 10.1097/HJH.0000000000003756 38747416

[B43] Han L. Ma C. Wu Z. Xu H. Li H. Pan G. (2024). AhR-STAT3-HO-1/COX-2 signalling pathway may restrict ferroptosis and improve hMSC accumulation and efficacy in mouse liver. Br. J. Pharmacol. 181, 125–141. 10.1111/bph.16208 37538043

[B44] Han W. Dong Z. Dimitropoulou C. Su Y. (2011). Hydrogen sulfide ameliorates tobacco smoke-induced oxidative stress and emphysema in mice. Antioxid. Redox Signal 15, 2121–2134. 10.1089/ars.2010.3821 21504365 PMC3166206

[B45] He Y. Z. Wang Y. X. Ma J. S. Li R. N. Wang J. Lian T. Y. (2023). MicroRNAs and their regulators: potential therapeutic targets in pulmonary arterial hypertension. Vasc. Pharmacol. 153, 107216. 10.1016/j.vph.2023.107216 37699495

[B46] Hennigan S. Channick R. N. Silverman G. J. (2008). Rituximab treatment of pulmonary arterial hypertension associated with systemic lupus erythematosus: a case report. Lupus 17, 754–756. 10.1177/0961203307087610 18625655

[B47] Hildebrandt T. M. Grieshaber M. K. (2008). Three enzymatic activities catalyze the oxidation of sulfide to thiosulfate in mammalian and invertebrate mitochondria. Febs J. 275, 3352–3361. 10.1111/j.1742-4658.2008.06482.x 18494801

[B48] Hou X. Y. Hu Z. L. Zhang D. Z. Lu W. Zhou J. Wu P. F. (2017). Rapid antidepressant effect of hydrogen sulfide: evidence for activation of mTORC1-TrkB-AMPA receptor pathways. Antioxid. Redox Signal 27, 472–488. 10.1089/ars.2016.6737 28158955

[B49] Hourihan J. M. Kenna J. G. Hayes J. D. (2013). The gasotransmitter hydrogen sulfide induces nrf2-target genes by inactivating the keap1 ubiquitin ligase substrate adaptor through formation of a disulfide bond between cys-226 and cys-613. Antioxid. Redox Signal 19, 465–481. 10.1089/ars.2012.4944 23145493

[B50] Hu B. Zhang X. X. Zhang T. Yu W. C. (2023). Dissecting molecular mechanisms underlying ferroptosis in human umbilical cord mesenchymal stem cells: role of cystathionine γ-lyase/hydrogen sulfide pathway. World J. Stem Cells 15, 1017–1034. 10.4252/wjsc.v15.i11.1017 38058959 PMC10696191

[B51] Hu L. F. Pan T. T. Neo K. L. Yong Q. C. Bian J. S. (2008). Cyclooxygenase-2 mediates the delayed cardioprotection induced by hydrogen sulfide preconditioning in isolated rat cardiomyocytes. Pflugers Arch. 455, 971–978. 10.1007/s00424-007-0346-8 17901979

[B52] Hu Q. Lukesh J. C. 3rd (2023). H(2)S donors with cytoprotective effects in models of MI/R injury and chemotherapy-induced cardiotoxicity. Antioxidants (Basel). 12, 650. 10.3390/antiox12030650 36978898 PMC10045576

[B53] Hu X. Lv X. Zhang L. Li S. S. Jin X. (2025). Noncoding rna lipotherapeutics: a promising breakthrough in pulmonary hypertension treatment. Curr. Pharm. Biotechnol. 26, 9–16. 10.2174/0113892010302590240321073509 38561610

[B54] Huang Y. Zhang H. Lv B. Tang C. Du J. Jin H. (2022). Sulfur dioxide: endogenous generation, biological effects, detection, and therapeutic potential. Antioxid. Redox Signal 36, 256–274. 10.1089/ars.2021.0213 34538110

[B55] Huerta De La Cruz S. Medina-Terol G. J. Tapia-Martínez J. A. Silva-Velasco D. L. Beltran-Ornelas J. H. Sánchez-López A. (2023). Hydrogen sulfide as a neuromodulator of the vascular tone. Eur. J. Pharmacol. 940, 175455. 10.1016/j.ejphar.2022.175455 36549499

[B56] Ichinose F. Hindle A. (2024). Sulfide catabolism in hibernation and neuroprotection. Nitric Oxide 146, 19–23. 10.1016/j.niox.2024.03.002 38521487 PMC11055667

[B57] Iciek M. Bilska-Wilkosz A. Kozdrowicki M. Górny M. (2022). Reactive sulfur species and their significance in health and disease. Biosci. Rep. 42. 10.1042/BSR20221006 PMC9484011 36039860

[B58] Iciek M. Kwiecień I. Włodek L. (2009). Biological properties of garlic and garlic-derived organosulfur compounds. Environ. Mol. Mutagen 50, 247–265. 10.1002/em.20474 19253339

[B59] Jackson M. R. Melideo S. L. Jorns M. S. (2012). Human sulfide:quinone oxidoreductase catalyzes the first step in hydrogen sulfide metabolism and produces a sulfane sulfur metabolite. Biochemistry 51, 6804–6815. 10.1021/bi300778t 22852582

[B60] Jaiswal A. K. Rajauria G. Abu-Ghannam N. Gupta S. (2011). Phenolic composition, antioxidant capacity and antibacterial activity of selected Irish Brassica vegetables. Nat. Prod. Commun. 6, 1299–1304. 10.1177/1934578x1100600923 21941903

[B61] Jiang Z. L. Liu Y. Zhang C. H. Chu T. Yang Y. L. Zhu Y. W. (2024). Emerging roles of hydrogen sulfide in colorectal cancer. Chem. Biol. Interact. 403, 111226. 10.1016/j.cbi.2024.111226 39237072

[B62] Jin H. F. Liang C. Liang J. M. Tang C. S. Du J. B. (2008). Effects of hydrogen sulfide on vascular inflammation in pulmonary hypertension induced by high pulmonary blood flow: experiment with rats. Zhonghua Yi Xue Za Zhi 88, 2235–2239. 19087668

[B63] Kabil O. Banerjee R. (2014). Enzymology of H2S biogenesis, decay and signaling. Antioxid. Redox Signal 20, 770–782. 10.1089/ars.2013.5339 23600844 PMC3910450

[B64] Kanemaru E. Ichinose F. (2025). Essential role of sulfide oxidation in brain health and neurological disorders. Pharmacol. Ther. 266, 108787. 10.1016/j.pharmthera.2024.108787 39719173 PMC11806942

[B65] Kang Y. Zhang G. Huang E. C. Huang J. Cai J. Cai L. (2020). Sulforaphane prevents right ventricular injury and reduces pulmonary vascular remodeling in pulmonary arterial hypertension. Am. J. Physiol. Heart Circ. Physiol. 318, H853–H866. 10.1152/ajpheart.00321.2019 32108526

[B66] Kao P. N. (2005). Simvastatin treatment of pulmonary hypertension: an observational case series. Chest 127, 1446–1452. 10.1378/chest.127.4.1446 15821229

[B67] Kherbeck N. Tamby M. C. Bussone G. Dib H. Perros F. Humbert M. (2013). The role of inflammation and autoimmunity in the pathophysiology of pulmonary arterial hypertension. Clin. Rev. Allergy Immunol. 44, 31–38. 10.1007/s12016-011-8265-z 21394427

[B68] Kim S. Kim D. B. Jin W. Park J. Yoon W. Lee Y. (2018). Comparative studies of bioactive organosulphur compounds and antioxidant activities in garlic (Allium sativum L.), elephant garlic (Allium ampeloprasum L.) and onion (Allium cepa L.). Nat. Prod. Res. 32, 1193–1197. 10.1080/14786419.2017.1323211 28475377

[B69] Lan D. Yao C. Li X. Liu H. Wang D. Wang Y. (2022). Tocopherol attenuates the oxidative stress of BMSCs by inhibiting ferroptosis through the PI3k/AKT/mTOR pathway. Front. Bioeng. Biotechnol. 10, 938520. 10.3389/fbioe.2022.938520 36061427 PMC9428255

[B70] Lawson L. D. Hunsaker S. M. (2018). Allicin bioavailability and bioequivalence from garlic supplements and garlic foods. Nutrients 10, 812. 10.3390/nu10070812 29937536 PMC6073756

[B71] Li K. X. Wang Z. C. Machuki J. O. Li M. Z. Wu Y. J. Niu M. K. (2022). Benefits of curcumin in the vasculature: a therapeutic candidate for vascular remodeling in arterial hypertension and pulmonary arterial hypertension? Front. Physiol. 13, 848867. 10.3389/fphys.2022.848867 35530510 PMC9075737

[B72] Li L. Li M. Li Y. Sun W. Wang Y. Bai S. (2016). Exogenous H2S contributes to recovery of ischemic post-conditioning-induced cardioprotection by decrease of ROS level via down-regulation of NF-κB and JAK2-STAT3 pathways in the aging cardiomyocytes. Cell. Biosci. 6, 26. 10.1186/s13578-016-0090-x 27096074 PMC4836181

[B73] Li L. Rose P. Moore P. K. (2011). Hydrogen sulfide and cell signaling. Annu. Rev. Pharmacol. Toxicol. 51, 169–187. 10.1146/annurev-pharmtox-010510-100505 21210746

[B74] Li M. Tang X. Liu X. Cui X. Lian M. Zhao M. (2020). Targeted miR-21 loaded liposomes for acute myocardial infarction. J. Mat. Chem. B 8, 10384–10391. 10.1039/d0tb01821j 33112352

[B75] Li Q. Lancaster J. R. Jr. (2013). Chemical foundations of hydrogen sulfide biology. Nitric Oxide 35, 21–34. 10.1016/j.niox.2013.07.001 23850631 PMC3843984

[B76] Li S. Yang G. (2015). Hydrogen sulfide maintains mitochondrial DNA replication via demethylation of TFAM. Antioxid. Redox Signal 23, 630–642. 10.1089/ars.2014.6186 25758951 PMC4554549

[B77] Li T. Li J. Li T. Zhao Y. Ke H. Wang S. (2021). L-cysteine provides neuroprotection of hypoxia-ischemia injury in neonatal mice via a PI3K/Akt-Dependent mechanism. Drug Des. Devel Ther. 15, 517–529. 10.2147/DDDT.S293025 PMC7886094 33603342

[B78] Li W. Jin H. F. Liu D. Sun J. H. Jian P. J. Li X. H. (2009). Hydrogen sulfide induces apoptosis of pulmonary artery smooth muscle cell in rats with pulmonary hypertension induced by high pulmonary blood flow. Chin. Med. J. Engl. 122, 3032–3038. 20137497

[B79] Li X. Du J. Jin H. Geng B. Tang C. (2008). Sodium hydrosulfide alleviates pulmonary artery collagen remodeling in rats with high pulmonary blood flow. Heart Vessels 23, 409–419. 10.1007/s00380-008-1059-4 19037589

[B80] Li X. X. Zeng J. Liu Y. Liang M. Liu Q. Li Z. (2020). Inhibitory effect and mechanism of action of quercetin and quercetin diels-alder anti-dimer on erastin-induced ferroptosis in bone marrow-derived mesenchymal stem cells. Antioxidants (Basel) 9, 205. 10.3390/antiox9030205 32131401 PMC7139729

[B81] Li X. H. Du J. B. Bu D. F. Tang X. Y. Tang C. S. (2006a). Sodium hydrosulfide alleviated pulmonary vascular structural remodeling induced by high pulmonary blood flow in rats. Acta Pharmacol. Sin. 27, 971–980. 10.1111/j.1745-7254.2006.00353.x 16867247

[B82] Li X. H. Du J. B. Tang C. S. (2006b). Impact of hydrogen sulfide donor on pulmonary vascular structure and vasoactive peptides in rats with pulmonary hypertension induced by high pulmonary blood flow. Zhongguo Yi Xue Ke Xue Yuan Xue Bao 28, 159–163. 16733895

[B83] Li Y. Liu G. Cai D. Pan B. Lin Y. Li X. (2014). H2S inhibition of chemical hypoxia-induced proliferation of HPASMCs is mediated by the upregulation of COX-2/PGI2. Int. J. Mol. Med. 33, 359–366. 10.3892/ijmm.2013.1579 24337227

[B84] Liao Y. X. Wang X. H. Bai Y. Lin F. Li M. X. Mi W. J. (2021). Relationship between endogenous hydrogen sulfide and pulmonary vascular indexes on high-resolution computed tomography in patients with chronic obstructive pulmonary disease. Int. J. Chron. Obstruct Pulmon Dis. 16, 2279–2289. 10.2147/COPD.S314349 34408410 PMC8364359

[B85] Libiad M. Vitvitsky V. Bostelaar T. Bak D. W. Lee H. J. Sakamoto N. (2019). Hydrogen sulfide perturbs mitochondrial bioenergetics and triggers metabolic reprogramming in colon cells. J. Biol. Chem. 294, 12077–12090. 10.1074/jbc.RA119.009442 31213529 PMC6690701

[B86] Liu A. J. Li B. Yang M. Liu Y. Liu Y. L. Su J. W. (2017). Sirtuin 1 mediates hydrogen sulfide-induced cytoprotection effects in neonatal mouse cardiomyocytes. Chin. Med. J. Engl. 130, 2346–2353. 10.4103/0366-6999.215328 28937042 PMC5634087

[B87] Liu J. J. Ren Z. Yang L. Zhu L. Li Y. Bie C. (2022). The NSUN5-FTH1/FTL pathway mediates ferroptosis in bone marrow-derived mesenchymal stem cells. Stem Cells 8, 99. 10.1038/s41420-022-00902-z PMC8898311 35249107

[B88] Liu M. Li Y. Liang B. Li Z. Jiang Z. Chu C. (2018). Hydrogen sulfide attenuates myocardial fibrosis in diabetic rats through the JAK/STAT signaling pathway. Int. J. Mol. Med. 41, 1867–1876. 10.3892/ijmm.2018.3419 29393353 PMC5810211

[B89] Liu M. Si Z. (2024). An update: epigenetic mechanisms underlying methamphetamine addiction. Front. Cell. Dev. Biol. 12, 1494557. 10.3389/fcell.2024.1494557 39650725 PMC11621221

[B90] Liu X. Zhou H. Zhang H. Jin H. He Y. (2023). Advances in the research of sulfur dioxide and pulmonary hypertension. Front. Pharmacol. 14, 1282403. 10.3389/fphar.2023.1282403 37900169 PMC10602757

[B91] Liu X. Y. Qian L. L. Wang R. X. (2022). Hydrogen sulfide-induced vasodilation: the involvement of vascular potassium channels. Front. Pharmacol. 13, 911704. 10.3389/fphar.2022.911704 35721210 PMC9198332

[B92] Liu Y. H. Lu M. Hu L. F. Wong P. T. Webb G. D. Bian J. S. (2012). Hydrogen sulfide in the mammalian cardiovascular system. Antioxid. Redox Signal 17, 141–185. 10.1089/ars.2011.4005 22304473

[B93] Lu F. Lu B. Zhang L. Wen J. Wang M. Zhang S. (2020). Hydrogen sulphide ameliorating skeletal muscle atrophy in db/db mice via Muscle RING finger 1 S-sulfhydration. J. Cell. Mol. Med. 24, 9362–9377. 10.1111/jcmm.15587 32633463 PMC7417732

[B94] Lv B. Chen S. Tang C. Jin H. Du J. Huang Y. (2021). Hydrogen sulfide and vascular regulation - an update. J. Adv. Res. 27, 85–97. 10.1016/j.jare.2020.05.007 33318869 PMC7728588

[B95] Madden J. A. Ahlf S. B. Dantuma M. W. Olson K. R. Roerig D. L. (2012). Precursors and inhibitors of hydrogen sulfide synthesis affect acute hypoxic pulmonary vasoconstriction in the intact lung. J. Appl. Physiol. (1985) 112 **,** 411–418. 10.1152/japplphysiol.01049.2011 22074719

[B96] Madonna R. Biondi F. Ghelardoni S. D'alleva A. Quarta S. Massaro M. (2024). Pulmonary hypertension associated to left heart disease: phenotypes and treatment. Eur. J. Intern Med. 129, 1–15. 10.1016/j.ejim.2024.07.030 39095300

[B97] Majtan T. Kožich V. Kruger W. D. (2023). Recent therapeutic approaches to cystathionine beta-synthase-deficient homocystinuria. Br. J. Pharmacol. 180, 264–278. 10.1111/bph.15991 36417581 PMC9822868

[B98] Majtan T. Krijt J. Sokolová J. Křížková M. Ralat M. A. Kent J. (2018). Biogenesis of hydrogen sulfide and thioethers by cystathionine beta-synthase. Antioxid. Redox Signal 28, 311–323. 10.1089/ars.2017.7009 28874062

[B99] Malliaras K. Marbán E. (2011). Cardiac cell therapy: where we've been, where we are, and where we should be headed. Br. Med. Bull. 98, 161–185. 10.1093/bmb/ldr018 21652595 PMC3149211

[B100] Mirhadi E. Mirhadi M. Kesharwani P. Sahebkar A. (2024). Therapeutic potential of organosulfur compounds in pulmonary hypertension. PharmaNutrition 27, 100382. 10.1016/j.phanu.2024.100382

[B101] Munteanu C. Popescu C. Vlădulescu-Trandafir A. I. Onose G. (2024). Signaling paradigms of H_2_S-induced vasodilation: a comprehensive review. A Compr. Rev. 13, 1158. 10.3390/antiox13101158 PMC11505308 39456412

[B102] Nicoleau S. Fellows A. Wojciak-Stothard B. (2021). Role of Krüppel-like factors in pulmonary arterial hypertension. Int. J. Biochem. Cell. Biol. 134, 105977. 10.1016/j.biocel.2021.105977 33839307

[B103] Nie X. Shen C. Tan J. Wu Z. Wang W. Chen Y. (2020). Periostin: a potential therapeutic target for pulmonary hypertension? Circ. Res. 127, 1138–1152. 10.1161/CIRCRESAHA.120.316943 32752980

[B104] Nishimura A. Tang X. Zhou L. Ito T. Kato Y. Nishida M. (2024). Sulfur metabolism as a new therapeutic target of heart failure. J. Pharmacol. Sci. 155, 75–83. 10.1016/j.jphs.2024.04.005 38797536

[B105] Olson K. R. (2018). H(2)S and polysulfide metabolism: conventional and unconventional pathways. Biochem. Pharmacol. 149, 77–90. 10.1016/j.bcp.2017.12.010 29248597

[B106] Olson K. R. Gao Y. Arif F. Arora K. Patel S. Deleon E. R. (2018). Metabolism of hydrogen sulfide (H(2)S) and production of reactive sulfur species (RSS) by superoxide dismutase. Redox Biol. 15, 74–85. 10.1016/j.redox.2017.11.009 29220697 PMC5725220

[B107] Olson K. R. Gao Y. Deleon E. R. Arif M. Arif F. Arora N. (2017). Catalase as a sulfide-sulfur oxido-reductase: an ancient (and modern?) regulator of reactive sulfur species (RSS). Redox Biol. 12, 325–339. 10.1016/j.redox.2017.02.021 28285261 PMC5350573

[B108] Olson K. R. Whitfield N. L. Bearden S. E. St Leger J. Nilson E. Gao Y. (2010). Hypoxic pulmonary vasodilation: a paradigm shift with a hydrogen sulfide mechanism. Am. J. Physiol. Regul. Integr. Comp. Physiol. 298, R51–R60. 10.1152/ajpregu.00576.2009 19889863 PMC2806212

[B109] Pan J. Wang R. Pei Y. Wang D. Wu N. Ji Y. (2023). Sulforaphane alleviated vascular remodeling in hypoxic pulmonary hypertension via inhibiting inflammation and oxidative stress. J. Nutr. Biochem. 111, 109182. 10.1016/j.jnutbio.2022.109182 36220525

[B110] Pan L. L. Qin M. Liu X. H. Zhu Y. Z. (2017). The role of hydrogen sulfide on cardiovascular homeostasis: an overview with update on immunomodulation. Front. Pharmacol. 8, 686. 10.3389/fphar.2017.00686 29018349 PMC5622958

[B111] Paul B. D. (2022). Cysteine metabolism and hydrogen sulfide signaling in Huntington's disease. Free Radic. Biol. Med. 186, 93–98. 10.1016/j.freeradbiomed.2022.05.005 35550919 PMC10066926

[B112] Paul B. D. Snyder S. H. (2012). H_2_S signalling through protein sulfhydration and beyond. Nat. Rev. Mol. Cell. Biol. 13, 499–507. 10.1038/nrm3391 22781905

[B113] Peng S. Zhao D. Li Q. Wang M. Zhang S. Pang K. (2022). Hydrogen sulfide regulates SERCA2a ubiquitylation via muscle RING finger-1 S-sulfhydration to affect cardiac contractility in db/db mice. Cells 11, 3465. 10.3390/cells11213465 36359861 PMC9658184

[B114] Pullamsetti S. S. Schermuly R. Ghofrani A. Weissmann N. Grimminger F. Seeger W. (2014). Novel and emerging therapies for pulmonary hypertension. Am. J. Respir. Crit. Care Med. 189, 394–400. 10.1164/rccm.201308-1543PP 24401129

[B115] Putnik P. Gabrić D. Roohinejad S. Barba F. J. Granato D. Mallikarjunan K. (2019). An overview of organosulfur compounds from Allium spp.: from processing and preservation to evaluation of their bioavailability, antimicrobial, and anti-inflammatory properties. Food Chem. 276, 680–691. 10.1016/j.foodchem.2018.10.068 30409648

[B116] Qi T. Xu F. Yan X. Li S. Li H. (2016). Sulforaphane exerts anti-inflammatory effects against lipopolysaccharide-induced acute lung injury in mice through the Nrf2/ARE pathway. Int. J. Mol. Med. 37, 182–188. 10.3892/ijmm.2015.2396 26531002

[B117] Qin Y. Qiao Y. Wang D. Li L. Li M. Yan G. (2022). Target nuclear factor erythroid 2-related factor 2 in pulmonary hypertension: molecular insight into application. Oxid. Med. Cell. Longev. 2022, 7845503. 10.1155/2022/7845503 35707273 PMC9192195

[B118] Qingyou Z. Junbao D. Weijin Z. Hui Y. Chaoshu T. Chunyu Z. (2004). Impact of hydrogen sulfide on carbon monoxide/heme oxygenase pathway in the pathogenesis of hypoxic pulmonary hypertension. Biochem. Biophys. Res. Commun. 317, 30–37. 10.1016/j.bbrc.2004.02.176 15047144

[B119] Rabinovitch M. Guignabert C. Humbert M. Nicolls M. R. (2014). Inflammation and immunity in the pathogenesis of pulmonary arterial hypertension. Circ. Res. 115, 165–175. 10.1161/CIRCRESAHA.113.301141 24951765 PMC4097142

[B120] Ried K. (2016). Garlic lowers blood pressure in hypertensive individuals, regulates serum cholesterol, and stimulates immunity: an updated meta-analysis and review. J. Nutr. 146, 389S–396S. 10.3945/jn.114.202192 26764326

[B121] Romeo L. Iori R. Rollin P. Bramanti P. Mazzon E. (2018). Isothiocyanates: an overview of their antimicrobial activity against human infections. Molecules 23, 624. 10.3390/molecules23030624 29522501 PMC6017699

[B122] Rose P. Dymock B. W. Moore P. K. (2015). GYY4137, a novel water-soluble, H2S-releasing molecule. Methods Enzymol. 554, 143–167. 10.1016/bs.mie.2014.11.014 25725521

[B123] Roubenne L. Marthan R. Le Grand B. Guibert C. (2021). Hydrogen sulfide metabolism and pulmonary hypertension. Cells 10, 1477. 10.3390/cells10061477 34204699 PMC8231487

[B124] Rudyk O. Aaronson P. I. (2021). Redox regulation, oxidative stress, and inflammation in group 3 pulmonary hypertension. Adv. Exp. Med. Biol. 1303, 209–241. 10.1007/978-3-030-63046-1_13 33788196

[B125] Sahu S. C. (2002). Dual role of organosulfur compounds in foods: a review. J. Environ. Sci. Health C Environ. Carcinog. Ecotoxicol. Rev. 20, 61–76. 10.1081/GNC-120005388 12734054

[B126] Salvi A. Bankhele P. Jamil J. M. Kulkarni-Chitnis M. Njie-Mbye Y. F. Ohia S. E. (2016). Pharmacological actions of hydrogen sulfide donors on sympathetic neurotransmission in the bovine anterior uvea, *in vitro* . Neurochem. Res. 41, 1020–1028. 10.1007/s11064-015-1784-x 26700431

[B127] Sanchez O. Sitbon O. Jaïs X. Simonneau G. Humbert M. (2006). Immunosuppressive therapy in connective tissue diseases-associated pulmonary arterial hypertension. Chest 130, 182–189. 10.1378/chest.130.1.182 16840400

[B128] Sánchez-Gloria J. L. Martínez-Olivares C. E. Rojas-Morales P. Hernández-Pando R. Carbó R. Rubio-Gayosso I. (2021). Anti-inflammatory effect of allicin associated with fibrosis in pulmonary arterial hypertension. Int. J. Mol. Sci. 22, 8600. 10.3390/ijms22168600 34445305 PMC8395330

[B129] Schiliro M. Bartman C. M. Pabelick C. (2021). Understanding hydrogen sulfide signaling in neonatal airway disease. Expert Rev. Respir. Med. 15, 351–372. 10.1080/17476348.2021.1840981 33086886 PMC10599633

[B130] Shi L. Du J. Pu D. Qi J. Wei B. Tang C. (2003). Effects of high pulmonary blood flow on pulmonary vascular structure and the gene expression of cystathionine-gamma-lyase. Beijing Da Xue Xue Bao Yi Xue Ban. 35, 566–570. 14710245

[B131] Sirmagul B. Ilgin S. Atli O. Usanmaz S. E. Demirel-Yilmaz E. (2013). Assessment of the endothelial functions in monocrotaline-induced pulmonary hypertension. Clin. Exp. Hypertens. 35, 220–227. 10.3109/10641963.2012.721838 22967272

[B132] Song Y. Cao S. Sun X. Chen G. (2024). The interplay of hydrogen sulfide and microRNAs in cardiovascular diseases: insights and future perspectives. Mamm. Genome 35, 309–323. 10.1007/s00335-024-10043-6 38834923

[B133] Song Y. Xu Z. Zhong Q. Zhang R. Sun X. Chen G. (2023). Sulfur signaling pathway in cardiovascular disease. Front. Pharmacol. 14, 1303465. 10.3389/fphar.2023.1303465 38074127 PMC10704606

[B134] Spassov S. G. Donus R. Ihle P. M. Engelstaedter H. Hoetzel A. Faller S. (2017). Hydrogen sulfide prevents formation of reactive oxygen species through PI3K/Akt signaling and limits ventilator-induced lung injury. Injury 2017, 3715037. 10.1155/2017/3715037 PMC5307128 28250891

[B135] Spiekerkoetter E. Tian X. Cai J. Hopper R. K. Sudheendra D. Li C. G. (2013). FK506 activates BMPR2, rescues endothelial dysfunction, and reverses pulmonary hypertension. J. Clin. Invest. 123, 3600–3613. 10.1172/JCI65592 23867624 PMC3726153

[B136] Su M. Wang J. Wang C. Wang X. Dong W. Qiu W. (2015). MicroRNA-221 inhibits autophagy and promotes heart failure by modulating the p27/CDK2/mTOR axis. Cell. Death Differ. 22, 986–999. 10.1038/cdd.2014.187 25394488 PMC4423182

[B137] Sun H. J. Hou B. Wang X. Zhu X. X. Li K. X. Qiu L. Y. (2016). Endothelial dysfunction and cardiometabolic diseases: role of long non-coding RNAs. Life Sci. 167, 6–11. 10.1016/j.lfs.2016.11.005 27838210

[B138] Sun H. J. Wang Z. C. Nie X. W. Bian J. S. (2022). Therapeutic potential of carbon monoxide in hypertension-induced vascular smooth muscle cell damage revisited: from physiology and pharmacology. Biochem. Pharmacol. 199, 115008. 10.1016/j.bcp.2022.115008 35318039

[B139] Sun X. Ku D. D. (2006). Allicin in garlic protects against coronary endothelial dysfunction and right heart hypertrophy in pulmonary hypertensive rats. Am. J. Physiol. Heart Circ. Physiol. 291, H2431–H2438. 10.1152/ajpheart.00384.2006 16731642

[B140] Sun X. Zhao D. Lu F. Peng S. Yu M. Liu N. (2020). Hydrogen sulfide regulates muscle RING finger-1 protein S-sulfhydration at Cys(44) to prevent cardiac structural damage in diabetic cardiomyopathy. Br. J. Pharmacol. 177, 836–856. 10.1111/bph.14601 30734268 PMC7024715

[B141] Sun Y. Y. Tang C. Jin H. Du J. (2022). Implications of hydrogen sulfide in development of pulmonary hypertension. Biomolecules 12, 772. 10.3390/biom12060772 35740897 PMC9221447

[B142] Tang C. Li X. Du J. (2006). Hydrogen sulfide as a new endogenous gaseous transmitter in the cardiovascular system. Curr. Vasc. Pharmacol. 4, 17–22. 10.2174/157016106775203144 16472173

[B143] Tangcharoen T. Ngernsritrakul T. Chandavimol M. Kamsorn C. Apakuppakul S. Yamwong S. (2024). Discordance between the European and the United States guideline criteria for atrial septal defect closure in adult patients with pulmonary hypertension and its clinical impact. Curr. Probl. Cardiol. 49, 102869. 10.1016/j.cpcardiol.2024.102869 39343052

[B144] Tao B. B. Zhu Q. Zhu Y. C. (2024). Mechanisms underlying the hydrogen sulfide actions: target molecules and downstream signaling pathways. Antioxid. Redox Signal. 40, 86–109. 10.1089/ars.2023.0401 37548532

[B145] Teoh J. P. Li X. Simoncini T. Zhu D. Fu X. (2020). Estrogen-mediated gaseous signaling molecules in cardiovascular disease. Trends Endocrinol. Metab. 31, 773–784. 10.1016/j.tem.2020.06.001 32682630

[B146] Thenappan T. Ormiston M. L. Ryan J. J. Archer S. L. (2018). Pulmonary arterial hypertension: pathogenesis and clinical management. Bmj 360, j5492. 10.1136/bmj.j5492 29540357 PMC6889979

[B147] Tian H. (2014). Advances in the study on endogenous sulfur dioxide in the cardiovascular system. Chin. Med. J. Engl. 127, 3803–3807. 10.3760/cma.j.issn.0366-6999.20133031 25382339

[B148] Tian Y. (2008). The role of gasotransmitters in the pathogenesis of hypoxic pulmonary hypertension. Zhongguo Dang Dai Er Ke Za Zhi 10, 98–101. 18289488

[B149] Toombs C. F. Insko M. A. Wintner E. A. Deckwerth T. L. Usansky H. Jamil K. (2010). Detection of exhaled hydrogen sulphide gas in healthy human volunteers during intravenous administration of sodium sulphide. Br. J. Clin. Pharmacol. 69, 626–636. 10.1111/j.1365-2125.2010.03636.x 20565454 PMC2883755

[B150] Turhan K. Alan E. Yetik-Anacak G. Sevin G. (2022). H(2)S releasing sodium sulfide protects against pulmonary hypertension by improving vascular responses in monocrotaline-induced pulmonary hypertension. Eur. J. Pharmacol. 931, 175182. 10.1016/j.ejphar.2022.175182 35940235

[B151] Verjans R. Peters T. Beaumont F. J. Van Leeuwen R. Van Herwaarden T. Verhesen W. (2018). MicroRNA-221/222 family counteracts myocardial fibrosis in pressure overload-induced heart failure. Hypertension 71, 280–288. 10.1161/HYPERTENSIONAHA.117.10094 29255073

[B152] Vitvitsky V. Kabil O. Banerjee R. (2012). High turnover rates for hydrogen sulfide allow for rapid regulation of its tissue concentrations. Antioxid. Redox Signal 17, 22–31. 10.1089/ars.2011.4310 22229551 PMC3342560

[B153] Vitvitsky V. Yadav P. K. Kurthen A. Banerjee R. (2015). Sulfide oxidation by a noncanonical pathway in red blood cells generates thiosulfate and polysulfides. J. Biol. Chem. 290, 8310–8320. 10.1074/jbc.M115.639831 25688092 PMC4375485

[B154] Wang R. Tang C. (2022). Hydrogen sulfide biomedical research in China-20 Years of hindsight. Antioxidants (Basel) 11, 2136. 10.3390/antiox11112136 36358508 PMC9686505

[B155] Wang R. R. Yuan T. Y. Wang J. M. Chen Y. C. Zhao J. L. Li M. T. (2022). Immunity and inflammation in pulmonary arterial hypertension: from pathophysiology mechanisms to treatment perspective. Pharmacol. Res. 180, 106238. 10.1016/j.phrs.2022.106238 35504356

[B156] Wang Y. Chen F. Zhang Y. Zheng X. Liu S. Tang M. (2022). Biphasic effect of sulforaphane on angiogenesis in hypoxia via modulation of both Nrf2 and mitochondrial dynamics. Food Funct. 13, 2884–2898. 10.1039/d1fo04112f 35179529

[B157] Wei H. L. Zhang C. Y. Jin H. F. Tang C. S. Du J. B. (2008). Hydrogen sulfide regulates lung tissue-oxidized glutathione and total antioxidant capacity in hypoxic pulmonary hypertensive rats. Acta Pharmacol. Sin. 29, 670–679. 10.1111/j.1745-7254.2008.00796.x 18501113

[B158] Wen Y. D. Zhu Y. Z. (2015). The pharmacological effects of S-Propargyl-Cysteine, a novel endogenous H2S-producing compound. Handb. Exp. Pharmacol. 230, 325–336. 10.1007/978-3-319-18144-8_16 26162842

[B159] Wołowiec Ł. Mędlewska M. Osiak J. Wołowiec A. Grześk E. Jaśniak A. (2023). MicroRNA and lncRNA as the future of pulmonary arterial hypertension treatment. Int. J. Mol. Sci. 24, 9735. 10.3390/ijms24119735 37298685 PMC10253568

[B160] Wu J. Pan W. Wang C. Dong H. Xing L. Hou J. (2019). H(2)S attenuates endoplasmic reticulum stress in hypoxia-induced pulmonary artery hypertension. Biosci. Rep. 39. 10.1042/BSR20190304 PMC6614575 31239370

[B161] Xiaohui L. Junbao D. Lin S. Jian L. Xiuying T. Jianguang Q. (2005). Down-regulation of endogenous hydrogen sulfide pathway in pulmonary hypertension and pulmonary vascular structural remodeling induced by high pulmonary blood flow in rats. Circ. J. 69, 1418–1424. 10.1253/circj.69.1418 16247221

[B162] Xie L. Gu Y. Wen M. Zhao S. Wang W. Ma Y. (2016). Hydrogen sulfide induces keap1 S-sulfhydration and suppresses diabetes-accelerated atherosclerosis via Nrf2 activation. Diabetes 65, 3171–3184. 10.2337/db16-0020 27335232 PMC8928786

[B163] Xu K. Wu F. Xu K. Li Z. Wei X. Lu Q. (2018). NaHS restores mitochondrial function and inhibits autophagy by activating the PI3K/Akt/mTOR signalling pathway to improve functional recovery after traumatic brain injury. Chem. Biol. Interact. 286, 96–105. 10.1016/j.cbi.2018.02.028 29567101

[B164] Xu Y. X. Wang Y. Y. Jia X. G. Wang Y. Shi L. Wang W. T. (2011). The relationship between endogenous hydrogen sulfide system and pulmonary hypertension induced by hypoxic hypercapnia. Zhongguo Ying Yong Sheng Li Xue Za Zhi 27, 300–304. 22097720

[B165] Yan J. H. Fan H. N. Ge R. L. (2008). Role of hydrogen sulfide in the hypoxia pulmonary hypertension. Sheng Li Ke Xue Jin Zhan 39, 359–361. 19119622

[B166] Yan X. Wu H. Wu Z. Hua F. Liang D. Sun H. (2017). The new synthetic H(2)S-releasing SDSS protects mc3t3-E1 osteoblasts against H(2)O(2)-induced apoptosis by suppressing oxidative stress, inhibiting MAPKs, and activating the PI3K/Akt pathway. Front. Pharmacol. 8, 07. 10.3389/fphar.2017.00007 28163684 PMC5247634

[B167] Yanfei W. Lin S. Junbao D. Chaoshu T. (2006). Impact of L-arginine on hydrogen sulfide/cystathionine-gamma-lyase pathway in rats with high blood flow-induced pulmonary hypertension. Biochem. Biophys. Res. Commun. 345, 851–857. 10.1016/j.bbrc.2006.04.162 16701554

[B168] Yang G. Wu L. Jiang B. Yang W. Qi J. Cao K. (2008). H2S as a physiologic vasorelaxant: hypertension in mice with deletion of cystathionine gamma-lyase. Science 322, 587–590. 10.1126/science.1162667 18948540 PMC2749494

[B169] Yang J. Minkler P. Grove D. Wang R. Willard B. Dweik R. (2019). Non-enzymatic hydrogen sulfide production from cysteine in blood is catalyzed by iron and vitamin B(6). Commun. Biol. 2, 194. 10.1038/s42003-019-0431-5 31123718 PMC6529520

[B170] Yang J. H. Gao J. E Y. Q. Jiao L. J. Wu R. Yan Q. Y. (2024). Hydrogen sulfide inhibits skeletal muscle ageing by up-regulating autophagy through promoting deubiquitination of adenosine 5'-monophosphate (AMP)-activated protein kinase α1 via ubiquitin specific peptidase 5. J. Cachexia Sarcopenia Muscle 15, 2118–2133. 10.1002/jcsm.13560 39189428 PMC11446701

[B171] Yang Y. Wang Y. Fan X. Xu X. Wang H. Wang X. (2025). Role of DNA methylation transferase in urinary system diseases: from basic to clinical perspectives (Review). Int. J. Mol. Med. 55, 19. 10.3892/ijmm.2024.5460 39575487 PMC11611324

[B172] Yang Y. Zhang B. K. Liu D. Nie W. Yuan J. M. Wang Z. (2012). Sodium hydrosulfide prevents hypoxia-induced pulmonary arterial hypertension in broilers. Br. Poult. Sci. 53, 608–615. 10.1080/00071668.2012.728284 23281754

[B173] Yi K. Guo T. Wang W. X. He S. E. Zhang X. Xu J. G. (2025). The relationship of nitric oxide synthase 3(NOS3) gene polymorphism in the risk of pulmonary arterial hypertension: a systematic review and meta-analysis. Nitric Oxide 154, 51–76. 10.1016/j.niox.2024.11.009 39580019

[B174] Yuan X. M. Zhuan B. Li P. Zhao X. Wang T. Yang Z. (2019). Expression of nicotinamide adenine dinucleotide phosphate-reduced oxidase-4/reactive oxygen species and cystathionine-γ-lyase/hydrogen sulfide in patients with chronic obstructive pulmonary disease-related pulmonary hypertension. Zhonghua Nei Ke Za Zhi 58, 770–776. 10.3760/cma.j.issn.0578-1426.2019.10.009 31594176

[B175] Zamanian R. T. Badesch D. Chung L. Domsic R. T. Medsger T. Pinckney A. (2021). Safety and efficacy of B-cell depletion with rituximab for the treatment of systemic sclerosis-associated pulmonary arterial hypertension: a multicenter, double-blind, randomized, placebo-controlled trial. Placebo-controlled Trial 204, 209–221. 10.1164/rccm.202009-3481OC PMC8650794 33651671

[B176] Zhang C. Du J. Bu D. Yan H. Tang X. Si Q. (2003). The regulatory effect of endogenous hydrogen sulfide on hypoxic pulmonary hypertension. Beijing Da Xue Xue Bao Yi Xue Ban. 35, 488–493. 14601305

[B177] Zhang D. Wang X. Chen S. Chen S. Yu W. Liu X. (2019). Endogenous hydrogen sulfide sulfhydrates IKKβ at cysteine 179 to control pulmonary artery endothelial cell inflammation. Clin. Sci. (Lond) 133, 2045–2059. 10.1042/CS20190514 31654061

[B178] Zhang D. Wang X. Tian X. Zhang L. Yang G. Tao Y. (2018). The increased endogenous sulfur dioxide acts as a compensatory mechanism for the downregulated endogenous hydrogen sulfide pathway in the endothelial cell inflammation. Front. Immunol. 9, 882. 10.3389/fimmu.2018.00882 29760703 PMC5936987

[B179] Zhang G. Kang Y. Cathey D. Leblanc A. J. Cai J. Cai L. (2022). Sulforaphane does not protect right ventricular systolic and diastolic functions in Nrf2 knockout pulmonary artery hypertension mice. Cardiovasc. Drugs Ther. 36, 425–436. 10.1007/s10557-022-07323-1 35157168 PMC9091145

[B180] Zhang H. Lin Y. Ma Y. Zhang J. Wang C. Zhang H. (2019). Protective effect of hydrogen sulfide on monocrotaline-induced pulmonary arterial hypertension via inhibition of the endothelial mesenchymal transition. Int. J. Mol. Med. 44, 2091–2102. 10.3892/ijmm.2019.4359 31573044 PMC6844600

[B181] Zhang J. Li Y. Chen Y. Zhang J. Jia Z. He M. (2024). o^8^G site-specifically modified tRF-1-AspGTC: a novel therapeutic target and biomarker for pulmonary hypertension. Hypertension 135, 76–92. 10.1161/CIRCRESAHA.124.324421 38747146

[B182] Zhang J. Li Y. Zhang J. Liu L. Chen Y. Yang X. (2023). ADAR1 regulates vascular remodeling in hypoxic pulmonary hypertension through N1-methyladenosine modification of circCDK17. Acta Pharm. Sin. B 13, 4840–4855. 10.1016/j.apsb.2023.07.006 38045055 PMC10692360

[B183] Zhang J. R. Sun H. J. (2020a). LncRNAs and circular RNAs as endothelial cell messengers in hypertension: mechanism insights and therapeutic potential. Mol. Biol. Rep. 47, 5535–5547. 10.1007/s11033-020-05601-5 32567025

[B184] Zhang J. R. Sun H. J. (2020b). Roles of circular RNAs in diabetic complications: from molecular mechanisms to therapeutic potential. Gene 763, 145066. 10.1016/j.gene.2020.145066 32827686

[B185] Zhang J. R. Sun H. J. (2021). MiRNAs, lncRNAs, and circular RNAs as mediators in hypertension-related vascular smooth muscle cell dysfunction. Hypertens. Res. 44, 129–146. 10.1038/s41440-020-00553-6 32985618

[B186] Zhang J. R. Sun H. J. (2022). Extracellular vesicle-mediated vascular cell communications in hypertension: mechanism insights and therapeutic potential of ncRNAs. Cardiovasc Drugs Ther. 36, 157–172. 10.1007/s10557-020-07080-z 32964302

[B187] Zhang L. Wang Y. Li Y. Li L. Xu S. Feng X. (2018). Hydrogen sulfide (H(2)S)-Releasing compounds: therapeutic potential in cardiovascular diseases. Front. Pharmacol. 9, 1066. 10.3389/fphar.2018.01066 30298008 PMC6160695

[B188] Zhang Q. Y. Du J. B. Shi L. Zhang C. Y. Yan H. Tang C. S. (2004). Interaction between endogenous nitric oxide and hydrogen sulfide in pathogenesis of hypoxic pulmonary hypertension. Beijing Da Xue Xue Bao Yi Xue Ban. 36, 52–56. 14970889

[B189] Zhang R. Shi W. Wu X. Yu Q. Xiao Y. (2025). Application of hydrogen sulfide donor conjugates in different diseases. Nitric Oxide 154, 128–139. 10.1016/j.niox.2024.11.008 39662602

[B190] Zhao H. Song J. Li X. Xia Z. Wang Q. Fu J. (2024). The role of immune cells and inflammation in pulmonary hypertension: mechanisms and implications. Front. Immunol. 15, 1374506. 10.3389/fimmu.2024.1374506 38529271 PMC10962924

[B191] Zhao M. Cheng Y. Wang X. Cui X. Cheng X. Fu Q. (2022). Hydrogen sulfide attenuates high-fat diet-induced obesity: involvement of mTOR/IKK/NF-κB signaling pathway. Mol. Neurobiol. 59, 6903–6917. 10.1007/s12035-022-03004-0 36053437

[B192] Zhong D. X. Zhang Y. Jin Q. Zhang X. C. Zhang F. Chen D. D. (2021). Increased serum PCSK9 in patients with idiopathic pulmonary arterial hypertension: insights from inflammatory cytokines. Pulm. Circ. 11, 20458940211051292. 10.1177/20458940211051292 34659741 PMC8516391

[B193] Zhu Y. W. Liu Z. T. Tang A. Q. Liang X. Y. Wang Y. Liu Y. F. (2024). The emerging roles of hydrogen sulfide in ferroptosis. Antioxid. Redox Signal. 41, 1150–1172. 10.1089/ars.2023.0535 39041626

